# Elucidating the role of key physio-biochemical traits and molecular network conferring heat stress tolerance in cucumber

**DOI:** 10.3389/fpls.2023.1128928

**Published:** 2023-02-20

**Authors:** Dhananjay A. Hongal, Dhandapani Raju, Sudhir Kumar, Akshay Talukdar, Anjan Das, Khushboo Kumari, Prasanta K. Dash, Viswanathan Chinnusamy, Anilabha Das Munshi, Tusar Kanti Behera, Shyam Sundar Dey

**Affiliations:** ^1^ Division of Vegetable Science, ICAR-Indian Agricultural Research Institute, New Delhi, India; ^2^ Division of Plant Physiology, ICAR-Indian Agricultural Research Institute, New Delhi, India; ^3^ Division of Genetics, ICAR-Indian Agricultural Research Institute, New Delhi, India; ^4^ ICAR-National Institute for Plant Biotechnology, New Delhi, India; ^5^ ICAR-Indian Institute of Vegetable Research, Varanasi, India

**Keywords:** cucumber, heat stress, physiological and biochemical traits, antioxidant enzymes, RT-PCR, HSPs

## Abstract

Cucumber is an important vegetable crop grown worldwide and highly sensitive to prevailing temperature condition. The physiological, biochemical and molecular basis of high temperature stress tolerance is poorly understood in this model vegetable crop. In the present study, a set of genotypes with contrasting response under two different temperature stress (35/30°C and 40/35°C) were evaluated for important physiological and biochemical traits. Besides, expression of the important heat shock proteins (HSPs), aquaporins (AQPs), photosynthesis related genes was conducted in two selected contrasting genotypes at different stress conditions. It was established that tolerant genotypes were able to maintain high chlorophyll retention, stable membrane stability index, higher retention of water content, stability in net photosynthesis, high stomatal conductance and transpiration in combination with less canopy temperatures under high temperature stress conditions compared to susceptible genotypes and were considered as the key physiological traits associated with heat tolerance in cucumber. Accumulation of biochemicals like proline, protein and antioxidants like SOD, catalase and peroxidase was the underlying biochemical mechanisms for high temperature tolerance. Upregulation of photosynthesis related genes, signal transduction genes and heat responsive genes (HSPs) in tolerant genotypes indicate the molecular network associated with heat tolerance in cucumber. Among the HSPs, higher accumulation of HSP70 and HSP90 were recorded in the tolerant genotype, WBC-13 under heat stress condition indicating their critical role. Besides, *Rubisco S, Rubisco L* and *CsTIP1b* were upregulated in the tolerant genotypes under heat stress condition. Therefore, the HSPs in combination with photosynthetic and aquaporin genes were the underlying important molecular network associated with heat stress tolerance in cucumber. The findings of the present study also indicated negative feedback of *G-protein alpha unit* and oxygen evolving complex in relation to heat stress tolerance in cucumber. These results indicate that the thermotolerant cucumber genotypes enhanced physio-biochemical and molecular adaptation under high-temperature stress condition. This study provides foundation to design climate smart genotypes in cucumber through integration of favorable physio-biochemical traits and understanding the detailed molecular network associated with heat stress tolerance in cucumber.

## Introduction

Agriculture and food safety are threatened by extreme climate change. High temperature (HT) stress restricts plant development and productivity and, in severe cases, even results in plant death (Bita and Gerats, 2013; Gong et al., 2020). Due to global warming, vegetable crops in tropical and subtropical areas, including tomato, pepper, and cucumber experienced decreased fruit number, weight, and shape throughout spring and autumn (Battisti and Naylor, 2009; Zhao et al., 2017). Abiotic stresses, such as drought stress on plants, will also be exacerbated by the high-temperature environment, which will also lead to the outbreak of several diseases (Cohen and Leach, 2020; Cohen et al., 2021). In particular, heat greatly affects plant growth and development, immunity and circadian rhythm, and poses a serious threat to the global food supply chain (Liu et al., 2015).

Cucumber is an annual vine crop is native to the Himalayan foothills (Wóycicki et al., 2011). India is the center of diversity for cultivated cucumber. Secondary centers of diversity for cucumber exist in China and the Near East (Meglic et al., 1996; Staub et al., 1999). Natural and artificial selection has contributed to the genetic differences observed between the cultivated and wild cucumber varieties (Wang et al., 2018). Cucumber is sensitive to high temperatures instead of its origin in tropical regions. The optimal temperature for its growth and development is 25-28°C during the day and 15-20°C at night (Tian et al., 2002). Cucumber plants in the early stage are susceptible to heat stress (HS) with the increasing global temperature, especially during the late spring and early autumn cultivation, where the temperature often exceeds 35°C (Tian et al., 2002). Besides, during the summer season, the temperature of cultivation in open fields often exceeds 35°C even during the seedling stage which leads to sunburn of leaves, growth retardation of stems and roots, and even plant death, which severely affects further growth of cucumber. In general, cucumber prefers a moderately warm environment, and the suitable temperature for growth is 20–30°C and at a temperature above 35°C abnormal growth is very common. Long-term high temperature above 40°C often results in metabolic malfunction, water loss and wilting of cucumber, and short-term extreme high temperature above 50°C leads to macro-molecule degradation, cell structure damage, dehydration and death, which has a great impact on the yield and quality of cucumber (Yu et al., 2018). Heat-related damage to cucumbers causes the blooms to fall easily, the leaves to droop and turn yellow, and the fruit to become malformed. In more extreme situations, the heat can cause the vine to completely wither, the top to die, the blooms to wither, and the leaves to get burnt and wilted. Thus, heat stress poses a significant threat for cucumber development, affecting production and quality during the summer. In the earlier studies, heat tolerance in the seedling stage was conducted using a limited number of genotypes, and studies were limited to the selected physiological traits like electrical conductivity, antioxidant enzymes, and chlorophyll estimation. Till date, no detailed studies regarding the physiological and biochemical basis of heat tolerance and the correlation of the heat stress response with the adult growth stage are reported.

Reactive oxygen species (ROS) are produced when high temperature (HT) is present, and these ROS cause membrane and pigment peroxidation, which reduces membrane permeability (Niu and Xiang, 2018). Additionally, HT changes the chloroplast and metabolite composition of leaves, which lowers the photosynthetic rate and causes plants to have a shorter lifespan and produce less (Djanaguiraman et al., 2018). The direct and indirect impacts of heat on sensor molecules located in many cellular components allow plants to detect heat stress. In response to heat stress, plants have evolved different avoidance and tolerance-based mechanisms. Heat avoidance includes all those strategies that plants adapt to avoid heat stress exposure while to survive under stressful conditions plants have evolved multiple of intrinsic tolerance mechanisms to adapt to high-temperature stress (Wang et al., 2016). To maintain life under high temperatures, plants have developed a variety of tolerance mechanisms. Physio-biochemical and molecular changes are important underlying mechanisms among the standard stress management techniques. Chlorophyll retention under stress is an effective way to sustain biomass production and crop yield (Wang et al., 2008). The ability to tolerate heat is demonstrated by a high membrane stability index and a high relative water content. To counteract the consequences of stress, it is crucial to have stress proteins, osmoprotectants, free-radical scavengers, ion transporters, and components involved in signaling cascades and transcriptional regulation (Wang et al., 2004). The understanding of various physiological, molecular, and biochemical pathways can facilitate the development of superior heat-tolerant genotypes in cucumber. Therefore, this study was conducted to address the aforementioned questions with objectives 1. To investigate the physio-biochemical basis for heat tolerance in cucumber. 2. Understanding the molecular networks for heat tolerance in cucumber for the key genes associated with heat stress tolerance.

## Material and methods

### Plant materials

**Figure 1:**
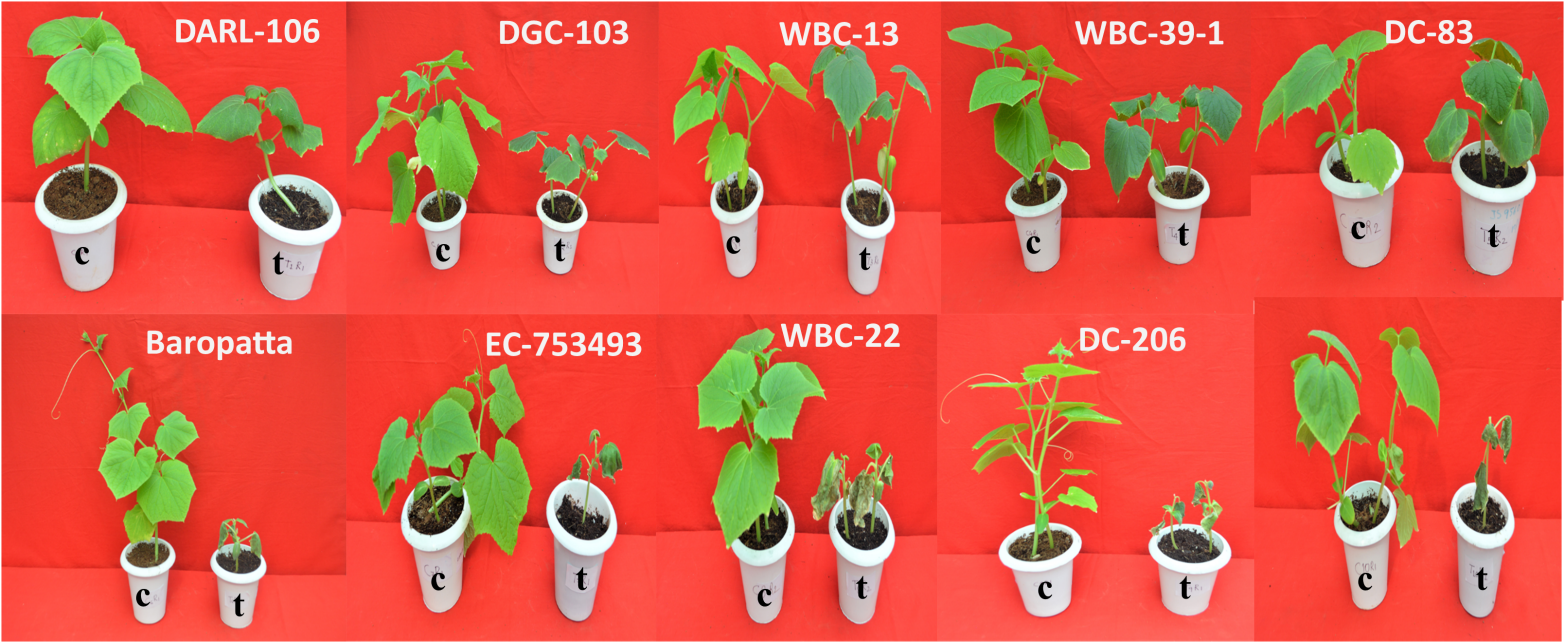
Heat stress response of the ten diverse cucumber genotypes under growth chamber with temperature stress treatment (40°C/35°C) along with controls (30°C/25°C); c-Control conditions, t-Treatment conditions (40°C/35°C).

The present experiment was carried out during 2021-22 at National phytotron facility, ICAR-Indian Agricultural Research Institute, New Delhi. The materials for present investigation comprised of 10 germplasms of cucumber collected from various parts of India. On the basis of their performance in two rounds of screening and performance under open field conditions, these genotypes are grouped into thermotolerant and thermosensitive. Five thermotolerant ‘TT’ and five thermosensitive ‘TS’ cucumber lines were grown in the pots with standard NPH potting mixture of soil, sand and coco peat in ratio 2:1:1 (v/v) (**Table 1**; **Figure 1**). Three seeds were sown in each pot and 5 replications were maintained for each genotype in both control and treatment conditions. A completely randomized design (CRD) with three replications per genotype per treatment was used. Data were taken from 3 randomly selected replications from each genotype. Seedlings were irrigated by sprayer cans with water and Hoagland nutrient solution every day morning and kept in such a condition that there was no water deficit. More frequent watering of plants was done under treatment to avoid any moisture stress. To avoid infections with fungal diseases, seedlings were occasionally sprayed with captan 2g/liter.

**Table 1:** List of the diverse set of genotypes expressed variable response to heat stress along with their key features.

	Genotype	Heat stress response	Salient feature	Yield under control conditions
1	DARL 106	Tolerant	Monoecious, non-parthenocarpic	2.4 Kg/Plant
2	DGC-103	Tolerant	Gynoecious non-parthenocarpic	2.8 Kg/Plant
3	WBC 13	Very Tolerant	Monoecious, non-parthenocarpic	2.0 Kg/Plant
4	WBC 39-1	Tolerant	Monoecious, non-parthenocarpic	2.0 Kg/Plant
5	DC83	Very Tolerant	Monoecious, non-parthenocarpic	3.0 kg/Plant
6	BAROPATTA	Sensitive	Monoecious, non-parthenocarpic	2.0 kg/Plant
7	EC-753493	Sensitive	Monoecious, non-parthenocarpic	2.0 kg/Plant
8	WBC 22	Sensitive	Monoecious, non-parthenocarpic	2.2 kg/Plant
9	DC-206	Sensitive	Monoecious, non-parthenocarpic	2.8 kg/Plant
10	DGPC-59	Very Sensitive	Predominantly gynoecious, parthenocarpic	2.6 kg/Plant

### High-temperature stress treatments

In control conditions plants were maintained at normal temperature (30°C/25°C day/night) in same glass house compartment throughout the experiments, while for treatment, plants were initially grown under normal conditions for twenty days, later seedlings were transferred to growing chamber for high temperature treatment. Therefore, the first HT treatment was set as 35°C for 12 h in the daytime and 30°C for 12 h in the night for 5 days, later growing chamber temperature raised to 40°C for 12 h in the daytime and 35°C for 12 h in the night for 5 days. Under the growth chamber, the plants were grown in growth media and were maintained without any moisture stress. Application of water and Hogland solution was practiced multiple times in a day to avoid water stress for the plants kept under temperature stress condition. Measurements were done on first or second true leaf of seedlings from three replications of each genotype in both control and treatment conditions. The physio-biochemical measurements were carried out at three different time intervals. First reading was recorded before transferring of the plants to growing chamber for treatment (day 0 control and day 0 stress). Remaining two readings were recorded after transferring of the plants to growing chamber for treatment. Second reading was noted on the last day of moderate stress (35°C/30°C) treatment (day 5 stress) and on same day readings were recorded under control conditions (day 5 control). Third reading was observed on the last day of high temperatures stress (40°C/35°C) treatment (day 10 stress) and on the same day data were recorded in control conditions (day 10 control).

### Chlorophyll measurement

The relative chlorophyll content of the leaves was measured by a SPAD chlorophyll meter (Apogee chlorophyll content meter). The measurements were done on the adaxial surface of the first and second true leaves in a single plant in five points uniformly distributed throughout the leaves and the average values were taken for analysis. The average value of two leaves was used to estimate the chlorophyll content. Chlorophyll was measured in CCI units. The CCI values of the instrument ranges from 1 to 100.

### Membrane stability index

Membrane stability index (MSI) of fresh leaves was determined as per the method suggested by Bailly et al. (1996). For this purpose, two plants of each genotype were randomly chosen per replicate and two leaf samples per plant were taken as follows. One sample from first true leaf and the second sample from second true leaf to represent mature and developing leaves, respectively. The conductivity of solutions was measured using a conductivity bridge meter and MSI calculated using following formulae:


$$\text{Membrane stability }\text{index MSI}=1-\frac{\text{C}1}{\text{C}2}$$

Where C1 conductivity at 40°C; C2 conductivity at 100°C

### Relative water content (RWC)

Leaf samples were used for Relative water content (RWC) assay according to the method described by Barrs and Weatherley, 1962. RWC in leaves of the plants was measured from two randomly chosen fully developed leaves. A 10 cm long segment was excised from the middle portion of the leaf and cut into two equal halves; FW was recorded and the leaf segments were immediately immersed into distilled water in a Petri plate for 4 h at room temperature. The leaf segments were blotted properly and turgid weight (TW) was recorded. Then, the samples were placed in a paper bag and dried in a hot air oven at 70°C for 24 h and the dry weight (DW) was recorded. The fresh weight (W1), turgid weight (W2), and dry weight (W3) of leaves were measured, and the RWC was calculated as follows:


$$\text{RWC}(\%)=\frac{\left(\text{W}1-\text{W}3\right)}{\left(\text{W}2-\text{W}3\right)}\times 100$$

### Photosynthesis and canopy temperature

Gas exchange measurements were performed using a portable photosynthesis system (Li-6400; LI-COR, Inc., Lincoln, NE, USA) in the morning between 9:00-10: 30am, on the first fully expanded leaf between 1 and 6 h of the light period on the third day of control (day 3 control), and moderate stress (day 3 stress) and high temperature treated (day 8 stress) plants. Air temperature was between 25°C - 30°C as per the temperature of the growth chamber. The light response curves were measured at ambient CO_2_ concentrations (350-400 μmol) during photosynthetic observations. Leaves were illuminated with photon flux densities 1500 μmol photons m^-2^s^-1^. Net photosynthetic rate (P_N_), stomatal conductance (g_s_), transpiration rate (E), and intracellular CO_2_ concentration (Ci) was measured. Canopy temperature of cucumber leaves was performed using an imaging FLUKE thermal imager.

### Morphological parameters

Morphological parameters like shoot length, fresh weight and dry weight, measurements were taken only once in both control and treatment conditions (10-day control and 10-day stress). Three plants from each genotype were for recording observations. Fresh weight and shoot length were measured immediately after harvesting of genotypes, whereas dry weight was measured by drying the samples in an oven at 85°C for two days.

### Biochemical parameters

#### Proline content

Proline (Pro) content was determined according to the protocol described previously by Bates et al. (1973). The second true leaf (0.5 g) were used to extract the proline, homogenized in 3% sulfosalicylic acid, and the supernatant was mixed with an equal volume of glacial acetic acid and acidic ninhydrin for the reaction. Following heating under 100°C for 30 min, a volume of 5 ml toluene was added to the mixture. The absorbance of the supernatant was measured at 520 nm using a UV–vis spectrometer and the standard curves which were made using l-proline in the same way. Proline activity is expressed as µmolg^-1^FW.

#### Super oxide dismutase

Super oxide dismutase content was determined according to the protocol described previously by Hwang et al. (1999). The activity of superoxide dismutase was measured by the ability of the enzyme to inhibit the light-dependent reduction of nitro blue tetrazolium chloride (NBT). The mixture was read at 560 nm and the amount of enzyme required to produce a 50% inhibition in the photoreduction rate of NBT was defined as one unit of SOD activity calculated as enzyme units (EU) per g of sample per minute (Ug^-1^FW min^-1^).

#### Catalase and guaiacol peroxidise

The catalase and guaiacol peroxidise activities were assayed as per the protocol of Pereira et al. (2002) and guaiacol peroxidase as per the protocol of Ramiro et al. (2006). CAT activity was measured by following the decomposition of H_2_O_2_ at 240 nm in a reaction mixture containing 50 mM phosphate buffer (pH 7.0) and 15 Mm H_2_O_2_. Enzyme activity was expressed as Ug^-1^FW For GPX, the oxidation of guaiacol was measured by following the increase in absorbance at 470 nm for 1 min. The assay mixture contained 50 mM phosphate buffer (pH 7.0), 0.1 mM EDTA, 10 mM guaiacol and 10 mM H_2_O_2_. GPOX activity was expressed as µmolg^-1^min^-1^

### MDA analysis

Determination of malonaldehyde (MDA) content was described by Dhindsa et al. (1981) and modified by Wang et al. (2018). A total of 0.5 g leaves were ground into powder using 0.5% trichloroacetic acid (TCA), then centrifuged at 3000g for 20 min. The supernatant (2 mL) was added the same volume of 0.5% thibabituric acid (TBA). After that the mixture was boiled at 100°C for 30 min to obtain the supernatants. Finally, we recorded the absorption wavelengths of supernatants on 450 532 and 600 nm. MDA activity was expressed as nmolg^-1^FW.

### Protein content

#### Protein quantification

Total soluble proteins were determined according to the method of Bradford (1976) with bovine serum albumin as a calibration standard. The homogenised leaf samples were used for preparation of the aliquot and estimation of protein. Protein content is expressed in terms of mgg^-1^.

### Ascorbate peroxidase content

Ascorbate peroxidase activity was determined according to Wang et al. (1991) by estimating the decreasing rate of ascorbate oxidation at 290 nm. APOD extraction was performed in 50 mM Tris–HCl (pH 7.2), 2% PVP, 1 mM EDTA, and 2 mM ascorbate. The reaction mixture consisted of 50 mM KH_2_2PO_4_ buffer (pH 6.6), 2.5 mM ascorbate, 10 mM H_2_O_2_, and enzyme, containing 100 µg proteins in a final volume of 1 mL. The enzyme activity was calculated from the initial rate of the reaction using the extinction coefficient of ascorbate (E = 2.8 mM cm−1 at 290 nm). APOX activity expressed in terms of µmol min^-1^g^-1^.

### Hydrogen peroxidase content

Hydrogen peroxidase contents were determined by the method Ohkawa et al. (1979). For determination of hydrogen peroxide, 0.5 mL of 0.1 M Tris–HCl (pH 7.6) and 1 mL of 1 M KI were added to 0.5 mL of supernatant. After 90 min, the absorbance was measured at 390 nm. A standard curve for hydrogen peroxide was prepared to calculate hydrogen peroxide concentration in each sample. Hydrogen peroxidase is expressed in terms of μmol g^-1^FW.

### RT-PCR analysis

Two contrasting genotypes, WBC-13 and DGPC-59 (**Supplementary Figure 1**) were used for gene expression analysis under two different stress conditions (35°C/30°C and 40°C/35°C) along with control without any stress (30°C/25°C). Total RNA from cucumber leaves under different stress conditions was extracted using Trizol reagent. RNA was quantified by spectrophotometric analysis and the quality was evaluated through agarose gel electrophoresis. First-strand complementary DNA (cDNA) synthesis was carried out using the user instruction (Promega, USA). Relative expression of 18 important genes associated with heat tolerance were conducted using two contrasting genotypes under two different stress conditions (**Table S1**). Quantitative real-time PCR was carried out using Light Cycler (Roche) with Light Cycler Fast Start DNA Master SYBR Green kit (Roche). Amplification of stress-related genes was carried out according to the manufacturer’s protocol. Reaction mixture of 20 μl contains 1.5 μl cDNA, 0.3 μl of primer (forward and reverse), 12.5 μl SYBR Premix, and 5.4 μl dH_2_O. Expression analysis of all genes were tested in triplicate with appropriate primers along with *Actin* used as an internal control. The gene expression data were calculated comparative to *Actin*, and Ct values of the used target genes were normalized using the Ct values of *Actin*. The levels of mRNA were also normalized with *Actin* and its value was expressed relative to that of the control, which was given an arbitrary value 1 (Liu et al., 2012). The relative differential gene expression was measured according to the equation 2−ΔΔCt (Livak and Schmittgen, 2001). The final data of RT-PCR were calculated from three experimental replicates.

### Statistical analysis

The data collected were analyzed in R software using one- or two-way ANOVA after verifying data for homogeneity and normality. The correlation among the variables was analyzed using Spearman correlation and a correlogram was constructed for each temperature treatment in the controlled heat-stress experiment and total plant responses from two stages for the field experiment. The multiple comparisons of means were made using Tukey’s HSD (Honestly Significant Difference) under α≤ 0.05. For only the significant main effects of stage, mean separation for the two stages were done within each level of varieties using Tukey’s HSD (Honestly Significant Difference) at α≤ 0.05.

## Results

Based on screening of genotypes under controlled environmental conditions and their validation under natural field conditions one set of 5 genotypes each in the tolerant and susceptible were selected for their detailed studies on important physiological and biochemical traits.

### Physiological basis of high temperature tolerance

#### Chlorophyll content

Chlorophyll content significantly decreased in all genotypes under heat stress treatments over the control (**Table 2**). However, TT genotypes were able to maintain chlorophyll content, shown decrease of 5.5% and 19.1% whereas susceptible genotypes shown higher chlorophyll degradation, shown decrease of 14.9% and 30.4% in moderate temperature treatment (35°C/30°C) and high temperature treatment (40°C/35°C), respectively. Among the genotypes, DC-83 (20.8 CCI units) and DGC-103 (17.7 CCI units) had highest chlorophyll at moderate stress condition. In peak stress condition, higher chlorophyll concentration was retained by DC-83 (18.1 CCI units) and DARL-106 (15. 3 CCI units) whereas DC-206 (11.0 CCI units, 7.6 CCI units) and EC-753493 (11.7 CCI units, 7.7 CCI units) shown lowest chlorophyll content at moderate as well as high temperature conditions (**Table 3**). This differential rate of decrease in chlorophyll content across cucumber genotypes showed the presence of genetic variability for chlorophyll retention under high temperature conditions. For chlorophyll concentration, genotype and genotype × temperature interaction effects were highly significant (p<0.05) (**Table S2**).

**Table 2:** Effect of high temperature treatment on important physiological parameters in cucumber.

Parameter	Tolerant group					Susceptible group				
	Control (mean)	35°C/30°C (mean)	Loss (%) at 35°C/30°C	40°C/35 (mean)	Loss (%) at 40°C/35°C	Control(mean)	35°C/30°C (mean)	Loss (%) at 35°C/30°C	40°C/35°C(mean)	Loss (%) at 40°C/35°C
Chlorophyll (CCI)	17.2	16.2***	5.8	13.9**	19.1	17.1	14.5**	14.9	11.9**	30.4
MSI (%)	81.7	76.0**	7.0	70.4**	13.8	82.7	62.6***	24.3	55.9***	32.5
RWC (%)	84.0	76.2**	9.4	73.3***	12.7	83.1	66.3***	20.2	61.3***	26.2
CFC	0.819	0.811***	0.9	0.806**	1.5	0.813	0.799*	1.8	0.774*	4.9

Significant at *p = 0.05, **p = 0.01 and ***p = 0.001.

**Table 3:** Evaluation of diverse genotypes for chlorophyll content, membrane stability Index, relative water content and canopy temperature at control, moderate and high temperature conditions.

		Chlorophyll content					Membrane stability index				
Sl.no	Genotype	Control (m ± SE)	35°C/30°C(m ± SE)	Loss (%) at 35°C/30°C	40°C/35°C(m ± SE)	Loss (%) at 40°C/35°C	Control (m ± SE)	35°C/30°C(m ± SE)	Loss (%) at 35°C/30°C	40°C/35°C(m ± SE)	Loss (%) at 40°C/35°C
1	DARL-106	17.7 ± 0.24^bc^	16.8 ± 1.57^abc^	4.9	15.3 ± 1.58^ab^	13.7	82.7 ± 0.36^a^	74.6 ± 0.82^a^	9.9	69.0 ± 0.35^bc^	16.6
2	DGC-103	18.7 ± 0.35^b^	17.7 ± 0.50^ab^	5.5	14.9 ± 0.74^abc^	20.3	79.6 ± 1.59^a^	74.5 ± 1.58^a^	6.4	70.8 ± 0.84^abc^	11.0
3	WBC-13	14.3 ± 0.17^fg^	13.4 ± 1.23^bcd^	5.8	12.0 ± 1.46^bcd^	16.1	83.0 ± 1.48^a^	78.5 ± 0.87^a^	5.4	73.7 ± 1.51^a^	11.2
4	WBC-39-1	13.6 ± 0.12^g^	12.3 ± 0.49^cd^	9.6	9.4 ± 0.29^cd^	30.9	82.6 ± 1.69^a^	75.8 ± 0.68^a^	8.2	66.4 ± 0.31^cd^	19.6
5	DC-83	21.8 ± 0.08^a^	20.8 ± 0.12^a^	4.6	18.1 ± 0.64^a^	17.1	80.4 ± 0.55^a^	76.2 ± 0.16^a^	5.1	71.8 ± 0.55^ab^	10.6
6	BAROPATTA	15.8 ± 0.25^de^	12.3 ± 0.27^cd^	21.9	9.8 ± 0.70^bcd^	38.2	82.5 ± 1.05^a^	60.2 ± 0.28^c^	27.1	59.9 ± 0.48^e^	27.4
7	EC-753493	17.0 ± 0.30^cd^	11.7 ± 0.53^cd^	31.4	7.7 ± 0.46^d^	54.9	82.4 ± 0.91^a^	67.9 ± 0.09^b^	17.6	53.1 ± 0.77^fg^	35.6
8	WBC-22	17.6 ± 0.20^bc^	14.3 ± 0.43^bcd^	18.6	11.7 ± 0.67^bcd^	33.7	84.4 ± 1.36^a^	65.8 ± 0.95^b^	22.0	54.1 ± 0.74^f^	35.9
9	DC-206	18.6 ± 0.17^b^	11.0 ± 1.44^d^	40.7	7.6 ± 1.31^d^	59.1	81.5 ± 0.01^a^	60.4 ± 1.26^c^	25.9	62.9 ± 0.37^de^	22.8
10	DGPC-59	15.6 ± 0.26^ef^	13.3 ± 0.19^bcd^	14.6	10.4 ± 0.78^bcd^	33.0	82.5 ± 1.12^a^	58.6 ± 0.60^c^	29.0	49.2 ± 0.38^g^	40.4
		Relative water content					Canopy temperature				
Sl.no	Genotype	Control	35°C/30°C	Loss (%) at 35°C/30°C	40°C/35°C	Loss (%) at 40°C/35°C	Control	35°C/30°C	CTD	40°C/35°C	CTD
1	DARL-106	85.9 ± 0.79^ab^	72.6 ± 0.89^bc^	15.5	71.5 ± 1.04^bcd^	16.7	28.1 ± 0.58^ab^	32.6 ± 0.15^cd^	2.3 ± 0.12	34.0 ± 0.37^b^	6.0 ± 0.30
2	DGC-103	85.9 ± 0.11^ab^	78.5 ± 0.57^a^	8.6	75.9 ± 0.19^ab^	11.7	27.9 ± 0.49^ab^	32.4 ± 0.22^d^	2.5 ± 0.18	35.8 ± 0.49^b^	4.2 ± 0.40
3	WBC-13	83.5 ± 0.89^ab^	79.4 ± 1.42^a^	4.9	76.3 ± 0.75^a^	8.7	26.8 ± 0.54^ab^	32.1 ± 0.03^d^	2.8 ± 0.03	34.2 ± 0.19^b^	5.8 ± 0.15
4	WBC-39-1	81.8 ± 0.24^bc^	72.3 ± 0.31^bc^	11.6	69.3 ± 0.59^cd^	15.2	29.1 ± 0.58^a^	32.9 ± 0.12^bcd^	2.1 ± 0.09	34.2 ± 0.47^b^	5.8 ± 0.38
5	DC-83	82.8 ± 0.95^bc^	77.7 ± 1.62^ab^	6.1	73.4 ± 0.19^abc^	11.3	27.6 ± 0.25^ab^	32.3 ± 0.31^d^	2.7 ± 0.25	34.3 ± 0.47^b^	5.7 ± 0.39
6	BAROPATTA	87.7 ± 0.07^a^	72.1 ± 1.09^ab^	17.7	67.2 ± 0.49^d^	23.3	26.7 ± 0.35^b^	34.4 ± 0.06^abc^	0.6 ± 0.05	38.3 ± 0.49^a^	1.7 ± 0.40
7	EC-753493	78.6 ± 0.20^c^	64.9 ± 0.91^de^	17.4	60.5 ± 0.44^ef^	23.0	27.2 ± 0.52^ab^	34.7 ± 0.09^ab^	0.3 ± 0.07	38.8 ± 0.84^a^	1.2 ± 0.68
8	WBC-22	82.9 ± 0.28^bc^	60.8 ± 0.21^e^	26.7	56.7 ± 0.80^f^	31.6	27.0 ± 0.71^ab^	34.6 ± 0.19^ab^	0.4 ± 0.15	39.7 ± 0.43^a^	0.3 ± 0.35
9	DC-206	83.4 ± 0.85^ab^	67.1 ± 0.66^cd^	19.5	62.6 ± 0.96^e^	25.0	27.1 ± 0.43^ab^	34.7 ± 0.09^ab^	0.3 ± 0.07	39.2 ± 0.59^a^	0.8 ± 0.48
10	DGPC-59	82.5 ± 0.84^bc^	63.1 ± 0.72^de^	23.5	59.5 ± 1.16^ef^	27.9	27.4 ± 0.40^ab^	34.9 ± 0.09^a^	0.1 ± 0.07	39.6 ± 0.36^a^	0.4 ± 0.29

Values within a group in a column bearing different letters are significantly different as determined by Tukey’s test.

#### Membrane stability index (MSI)

Significant genetic variability in high temperature induced electrolyte leakage was also observed in cucumber genotypes taken for study. Tolerant genotypes showed slight decrease in MSI under heat stress condition, whereas drastic reduction in MSI was recorded in case of sensitive genotypes under temperature stress condition (**Table 2**). MSI decreased by 7.0% and 13.8% in tolerant group in contrast to 24.3% and 32.5% in susceptible group under moderate and high treatment conditions, respectively. In **Table 3**, it was depicted that at 35°C/30°C treatment and 40°C/35°C treatment, highest membrane stability was observed WBC-13 (78.5%,73.7%) and DC-83 (76.2%, 71.8%) whereas minimum in DGPC-59 (58.6%) followed by Baropatta (60.2%) at moderate stress conditions and DGPC-59 (49.2%) followed by EC-753493 (53.1%) at high stress conditions. Genotype, temperature and genotype × temperature interaction had an impact on membrane stability and significant effects were observed (**Table S2**).

#### Relative water content

In cucumber seedlings, RWC was measured under different heat stress conditions. Under stress combination, RWC levels showed a significant decrease as compared to control (**Table 2**). Heat stress resulted drastic reduction in RWC in all susceptible genotypes compared to tolerant genotypes. RWC levels decreased by 9.4% and 12.7% in tolerant group whereas RWC decreased by 20.2% and 26.2% in susceptible group under heat treatment over the control. It was depicted that under 35°C/30°C and 40°C/35°C treatments, highest tension of relative water content was observed WBC-13 (79.4%,76.3%) and DGC-103 (78.5%, 75.9%) whereas minimum water content was seen in WBC-22 (60.8%, 56.7%) followed by DGPC-59 (63.1%, 59.5%) at moderate and high temperature stress conditions, respectively (**Table 3**). The effects of genotype, temperature, and genotype × temperature interaction on RWC were presented in **Table S1** and were significant (p<0.05).

### Canopy temperature

**Figure 2:**
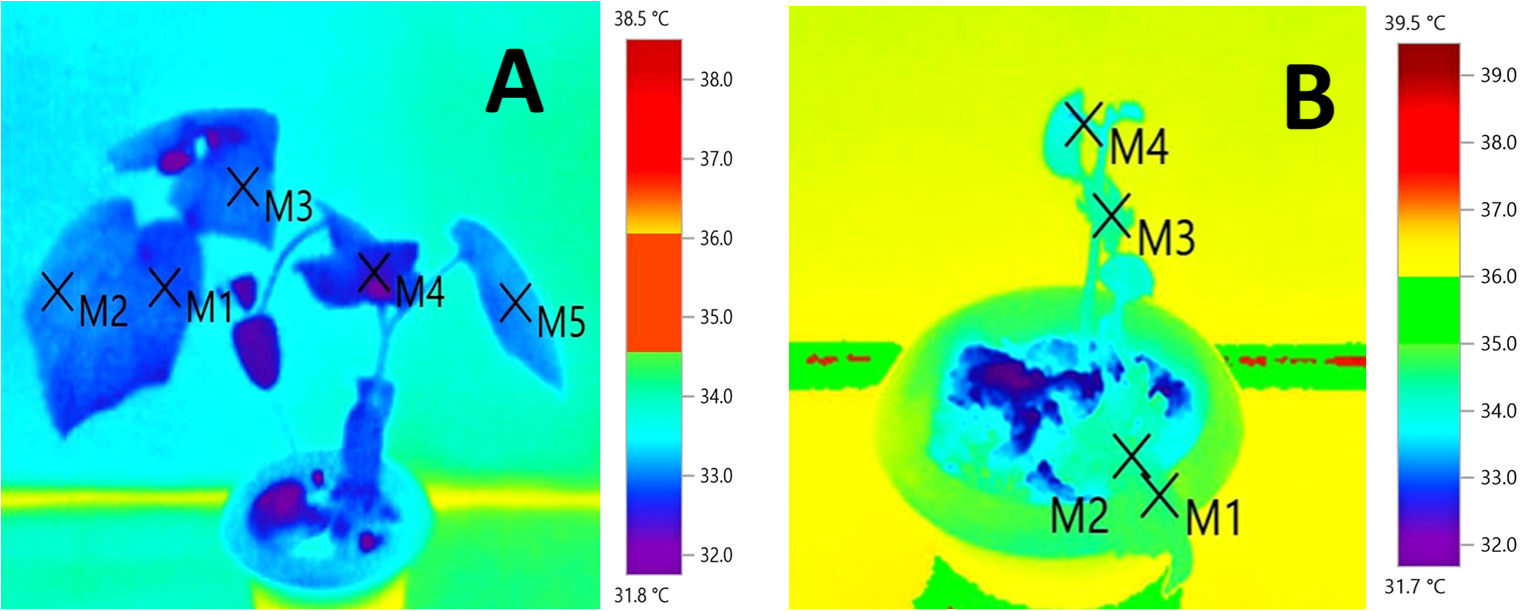
Infrared thermography of the thermotolerant cucumber genotype, WBC-13 **(A)** along with susceptible genotype, DGPC-59 **(B)** at high stress conditions 40°C/35°C.

It was evident that the tolerant genotypes were maintaining comparatively less canopy temperature by transpirational cooling than the susceptible genotypes in high temperature treatment conditions (**Figure 2**; **Supplementary Figure 2**). Lowest canopy temperature was recorded in the tolerant genotypes WBC-13, DGC-103 and DC-83 at moderate temperature stress and the same of set of genotypes also maintained a comparatively lowerer canopy temperature under high temperature stress condition (**Table 3**). The effects of genotypes, temperature, and genotype × temperature interaction effects on canopy temperature were significant (**Table S2**).

### Photosynthetic rate and leaf gas exchange related parameters

Leaf Pn, Gs, Ci, and Tr were significantly decreased on exposure to heat stress conditions in susceptible genotypes compared to tolerant genotypes. The net photosynthesis of susceptible genotypes was significantly lower than the tolerant genotypes under temperature stress conditions (**Supplementary Figure 3A**). Percentage decrease of Pn was 29.4% and 67.7% in susceptible groups in contrast to 7.9% and 24.1% in tolerant group under heat stress conditions (**Table 4**). Similarly, stomatal conductance (Gs) of all genotypes decreased to varied extent at high temperature treatments compared to the controls. Tolerant genotypes expressed higher stomatal conductance compared to susceptible genotypes under heat stress conditions (**Supplementary Figure 3B**). Reduction in stomatal conductance was 23.6% and 37.7% in tolerant group and 55.8% and 80.2% among the susceptible genotypes (**Table 4**). Tolerant genotypes recorded higher internal C*O*_2_ concentration (Ci) at high temperature treatments compared to the susceptible genotypes (**Supplementary Figure 4A**). Tolerant plants shown less reduction in Ci percentage (29.5% and 46.1%) as compared to drastic reduction in susceptible genotypes (37.7% and 58.9%) under stress conditions (**Table 4**). Transpiration rate (Tr) was significantly reduced at high temperature treatment in susceptible genotypes compared to tolerant genotypes (**Supplementary Figure 4B**). High temperature adversely affected transpiration rate of susceptible genotypes (55.5% and 66.3% reduction) compared to tolerant varieties (15.36% and 14.87%) as shown in **Table 4**.

**Table 4:** Effect of high temperature treatment on photosynthetic and gaseous parameters in cucumber genotypes.

Parameter	Tolerant group					Susceptible group				
	Control (mean)	35°C/30°C (mean)	Loss (%) at 35°C/30°C	40°C/35 (mean)	Loss (%) at 40°C/35°C	Control(mean)	35°C/30°C (mean)	Loss (%) at 35°C/30°C	40°C/35°C(mean)	Loss (%) at 40°C/35°C
Pn	18.7	17.2*	7.9	14.2*	24.1	19.5	13.7**	29.4	6.3**	67.7
Gs	0.691	0.528*	23.6	0.431*	37.7	0.685	0.303*	55.8	0.136*	80.2
Ci	763.9	538.4***	29.5	412.0***	46.1	739.6	460.8***	37.7	304.3***	58.9
Tr	5.0	4.2**	15.3	4.2**	14.8	6.8	3.0*	55.5	2.3*	66.3

Significant at *p = 0.05, **p = 0.01 and ***p = 0.001.

The tolerant genotype, DC-83 (19.2 μmol C*O*_2_ *m* ^-2^ *s* ^-1^, 16.1 μmol C*O*_2_ *m* ^-2^ *s* ^-1^) and WBC-13(18.3 μmol C*O*_2_ *m* ^-2^ *s* ^-1^, 16 μmol C*O*_2_ *m* ^-2^ *s* ^-1^) had stable net photosynthesis in moderate and high temperature stress conditions, respectively. Drastic reduction in photosynthesis was recorded in Baropatta (12.7 μmol C*O*_2_ *m* ^-2^ *s* ^-1^), WBC-22 (13.2 μmol C*O*_2_ *m* ^-2^ *s* ^-1^) at moderate heat stress conditions and DGPC-59 (4.5 μmol C*O*_2_ *m* ^-2^ *s* ^-1^) and EC-753493 (5.1 μmol C*O*_2_ *m* ^-2^ *s* ^-1^) in peak stress conditions. Maximum stomatal conductance was observed in DC-83 (0.57 mol *H*_2_ *Om* ^-2^ *s* ^-1^) followed by WBC-13(0.57 mol *H*_2_ *Om* ^-2^ *s* ^-1^) and minimum was seen in DGPC-59 (0.24 mol *H*_2_ *Om* ^-2^ *s* ^-1^), followed by DC-206 (0.25 mol *H*_2_ *Om* ^-2^ *s* ^-1^) in moderate stress. Similarly, WBC-13 (0.53 mol *H*_2_ *Om* ^-2^ *s* ^-1^) and DC-83 (0.49 mol *H*_2_ *Om* ^-2^ *s* ^-1^) had high stomatal conductance where as drastic drop in stomatal conductance was recorded in DGPC-59 (0.08 mol *H*_2_ *Om* ^-2^ *s* ^-1^) and Baropatta (0.11 mol *H*_2_ *Om* ^-2^ *s* ^-1^) at peak stress conditions (**Table 5**).

**Table 5:** Evaluation of ten diverse genotypes for Net Photosynthesis, Stomatal Conductance, Internal CO_2_ concentration and Transpiration rate under control, moderate and high temperature conditions.

		Net Photosynthesis					Stomatal Conductance				
Sl.no	Genotype	Control(m ± SE)	35°C/30°C(m ± SE)	Loss (%) at 35°C/30°C	40°C/35°C(m ± SE)	Loss (%) at 40°C/35°C	Control(m ± SE)	35°C/30°C(m ± SE)	Loss (%) at 35°C/30°C	40°C/35°C(m ± SE)	Loss (%) at 40°C/35°C
1	DARL-106	16.9 ± 0.23^cd^	15.5 ± 0.12^de^	3.5	13.5 ± 0.02^b^	16.2	0.83 ± 0.07^b^	0.51 ± 0.03^a^	38.4	0.40 ± 0.001^b^	51.3
2	DGC-103	17.1 ± 0.36^cd^	15.8 ± 0.03^cd^	7.3	12.5 ± 0.03^b^	26.4	0.66 ± 0.02^bcd^	0.47 ± 0.02^a^	28.1	0.42 ± 0.007^ab^	34.4
3	WBC-13	18.6 ± 0.01^b^	18.3 ± 0.46^a^	1.6	15.9 ± 0.40^a^	14.2	0.67 ± 0.105^bcd^	0.56 ± 0.01^a^	15.8	0.53 ± 0.008^a^	20.1
4	WBC-39-1	21.0 ± 0.05^a^	16.9 ± 0.12^bc^	19.6	12.6 ± 0.43^b^	39.8	0.61 ± 0.04^bcd^	0.50 ± 0.004^a^	25.2	0.27 ± 0.007^c^	60.2
5	DC-83	19.2 ± 0.19^b^	17.3 ± 0.58^ab^	5.6	16.0 ± 0.58^a^	21.1	0.60 ± 0.01^cd^	0.57 ± 0.005^a^	5.0	0.49 ± 0.004^ab^	16.8
6	BAROPATTA	17.6 ± 0.23^c^	12.6 ± 0.04^g^	28.2	7.9 ± 0.45^c^	55.2	1.36 ± 0.04^a^	0.36 ± 0.002^a^	73.2	0.11 ± 0.004^de^	92.3
7	EC-753493	21.9 ± 0.02^a^	13.3 ± 0.06^fg^	39.0	5.0 ± 0.05^ef^	76.8	0.79 ± 0.02^bc^	0.26 ± 0.001^a^	67.1	0.20 ± 0.003^cd^	75.3
8	WBC-22	16.4 ± 0.17^d^	13.2 ± 0.07^fg^	19.6	7.7 ± 0.07^cd^	53.2	0.49 ± 0.001^de^	0.38 ± 0.005^a^	22.4	0.18 ± 0.001^cde^	66.0
9	DC-206	19.3 ± 0.06^b^	14.4 ± 0.17^ef^	25.3	6.2 ± 0.08^de^	67.9	0.35 ± 0.009^e^	0.25 ± 0.005^a^	27.8	0.22 ± 0.001^c^	64.1
10	DGPC-59	21.9 ± 0.02^a^	14.9 ± 0.04^de^	31.8	4.4 ± 0.004^f^	79.5	0.61 ± 0.001^bcd^	0.24 ± 0.002^a^	41.1	0.08 ± 0.004^e^	80.7
		Internal CO_2_ Concentration					Transpiration Rate				
Sl.no	Genotype	Control(m ± SE)	35°C/30°C(m ± SE)	Loss (%) at 35°C/30°C	40°C/35°C(m ± SE)	Loss (%) at 40°C/35°C	Control(m ± SE)	35°C/30°C(m ± SE)	Loss (%) at 35°C/30°C	40°C/35°C(m ± SE)	Loss (%) at 40°C/35°C
1	DARL-106	767.2 ± 4.38^ab^	548.3 ± 11.88^ab^	28.5	372.9 ± 0.02^f^	51.4	4.7 ± 0.002^cd^	4.1 ± 0.03^abc^	13.6	4.1 ± 0.01^b^	12.3
2	DGC-103	772.1 ± 1.26^a^	533.1 ± 7.68^bc^	31.0	413.6 ± 0.30^b^	46.4	4.1 ± 0.04^d^	3.8 ± 0.13^bcd^	7.5	3.5 ± 0.01^c^	13.9
3	WBC-13	760.4 ± 6.08^bc^	526.2 ± 5.38^bc^	30.8	490.6 ± 5.07^a^	35.5	5.4 ± 0.01^bcd^	4.5 ± 0.15^ab^	15.9	4.7 ± 0.01^a^	13.5
4	WBC-39-1	753.6 ± 0.14^cd^	525.0 ± 1.31^c^	30.3	401.7 ± 0.60^c^	46.7	4.8 ± 0.38^cd^	3.8 ± 0.38^bcd^	20.6	4.2 ± 0.01^b^	13.4
5	DC-83	766.2 ± 0.48^ab^	558.9 ± 0.36^a^	27.1	381.1 ± 0.18^e^	50.3	5.9 ± 0.01^abcd^	4.8 ± 0.03^a^	17.6	4.7 ± 0.01^a^	20.0
6	BAROPATTA	774.9 ± 2.37^a^	488.0 ± 4.47^d^	37.0	391.4 ± 0.37^d^	49.5	7.7 ± 0.83^ab^	3.7 ± 0.17^bcd^	51.7	2.2 ± 0.01^e^	70.3
7	EC-753493	769.0 ± 3.51^ab^	477.3 ± 1.06^de^	37.9	303.6 ± 1.25^g^	60.5	6.6 ± 0.57^abc^	3.1 ± 0.07^de^	53.0	3.1 ± 0.03^d^	53.1
8	WBC-22	747.4 ± 0.69^d^	457.4 ± 2.93^e^	38.8	260.4 ± 0.55^j^	65.2	7.8 ± 0.48^a^	2.3 ± 0.02^e^	70.1	1.8 ± 0.01^f^	77.0
9	DC-206	688.4 ± 0.45^f^	471.6 ± 7.16^de^	31.5	293.5 ± 0.34^h^	57.4	7.5 ± 1.42^ab^	3.5 ± 0.42^cd^	53.0	2.3 ± 0.01^e^	69.3
10	DGPC-59	718.0 ± 0.84^e^	409.7 ± 0.91^f^	42.9	272.3 ± 0.53^i^	62.1	4.4 ± 0.01^cd^	2.4 ± 0.01^e^	44.7	1.9 ± 0.01^ef^	55.4

Values within a group in a column bearing different letters are significantly different as determined by Tukey’s test.

Maximum internal C*O*_2_ concentration was observed in DC-83 (558.9 µmolC*O*_2_ *mol* ^-1^) followed by DARL 106 (548.9 µmolC*O*_2_ *mol* ^-1^) and minimum was recorded in DGPC-59 (407.9 µmolC*O*_2_ *mol* ^-1^), followed by WBC-22 (457.4 µmolC*O*_2_ *mol* ^-1^) in moderate stress. WBC-13 (490.9 µmolC*O*_2_ *mol* ^-1^) and DGC-103 (413.0 µmolC*O*_2_ *mol* ^-1^) had high internal C*O*_2_ concentration where as drastic drop in stomatal conductance was recorded in DGPC-59 (272.3 µmolC*O*_2_ *mol* ^-1^) and WBC-22 (260.4 µmolC*O*_2_ *mol* ^-1^) at peak stress conditions. Similarly, highest transpiration rate was recorded in DC-83 (4.8 m mol *H*_2_O*m* ^-2^ *s* ^-1^) followed by WBC-13 (4.5 m mol *H*_2_O*m* ^-2^ *s* ^-1^) and minimum was recorded in WBC-22 (2.3 m mol *H*_2_O*m* ^-2^ *s* ^-1^) followed by DGPC-59 (2.4 m mol *H*_2_O*m* ^-2^ *s* ^-1^) in moderate stress and WBC-13 (4.7 m mol *H*_2_O*m* ^-2^ *s* ^-1^), DC 83 (4.7 m mol *H*_2_O*m* ^-2^ *s* ^-1^) shown highest transpiration rate whereas WBC-22 (1.8 m mol *H*_2_O*m* ^-2^ *s* ^-1^) followed by DGPC-59 (1.9 m mol *H*_2_O*m* ^-2^ *s* ^-1^) had lowest transpiration rate at peak stress conditions (**Table 5**).

Genotype, temperature and genotype × temperature interaction had an impact on photosynthetic pigment and leaf gas exchange−related parameters and significant effects were observed (**Table S3**).

### Morphological characteristics

Shoot length, fresh weight and dry weight of all genotypes as well as percentage change are presented in **Table 6**. Fresh weight, shoot length and dry weight were significantly reduced in seedlings grown under high temperature stress condition in all the genotypes. However, tolerant group showed less reduction (32.1%) in shoot length whereas higher reduction was recorded in susceptible group (71.7%) under high temperature stress condition. Significant reduction was also observed in fresh weight in both tolerant and susceptible genotypes. But tolerant genotypes were able to maintain the fresh weight in treatment conditions in contrast to susceptible genotypes. Reduction in fresh weight was higher in susceptible group of genotypes (87.0%) whereas in tolerant group shown much lower (62.7%) reduction in treatment conditions. Significant reduction in dry weight was recorded in all genotypes, however, percent of reduction was high in susceptible genotypes (67.4%) compared tolerant group (36.3%) under heat stress conditions.

**Table 6:** Effect of high temperature on important morphological characteristics in cucumber genotypes.

Shoot length					Fresh weight			Dry weight		
Tolerant Genotype		Control (m ± SE)	Treatment (m ± SE)	%Reduction	Control (m ± SE)	Treatment (m ± SE)	%Reduction	Control (m ± SE)	Treatment (m ± SE)	%Reduction
DARL 106		21.6 ± 0.45	15.3 ± 0.56	29.3	40.0 ± 0.26	12.9 ± 0.09	67.7	2.3 ± 0.05	1.3 ± 0.01	42.7
DGC-103		23.2 ± 4.56	14.1 ± 0.73	39.4	50.5 ± 0.17	15.4 ± 0.22	69.4	2.7 ± 0.01	1.6 ± 0.02	38.8
WBC 13		28.0 ± 0.16	20.0 ± 0.94	28.5	45.4 ± 0.83	21.1 ± 0.20	53.3	4.1 ± 0.01	2.5 ± 0.27	37.5
WBC 39-1		23.3 ± 1.19	14.6 ± 0.54	37.1	42.9 ± 0.42	14.6 ± 0.18	65.8	2.6 ± 0.05	1.8 ± 0.13	30.5
DC83		12.6 ± 0.76	9.3 ± 0.49	26.3	21.9 ± 0.12	10.5 ± 0.05	51.7	1.6 ± 0.02	1.1 ± 0.04	29.1
Average		21.7	14.6**	32.1	40.1	14.9**	62.7	2.6	1.7**	36.3
Susceptible Genotype		Control(m ± SE)	Treatment (m ± SE)	%Reduction	Control(m ± SE)	Treatment (m ± SE)	%Reduction	Control(m ± SE)	Treatment (m ± SE)	%Reduction
BAROPATTA		52.7 ± 0.29	11.2 ± 0.39	78.7	62.5 ± 0.40	7.6 ± 0.10	87.7	1.2 ± 0.02	0.5 ± 0.01	54.3
EC-753493		26.3 ± 1.91	10.1 ± 0.72	61.3	32.1 ± 0.11	6.5 ± 0.10	79.7	1.3 ± 0.02	0.4 ± 0.01	64.3
WBC 22		17.5 ± 1.50	10.0 ± 0.19	42.5	35.5 ± 0.54	7.8 ± 0.06	78.0	2.3 ± 0.01	0.2 ± 0.01	88.3
DC-206		48.6 ± 8.31	8.7 ± 0.53	81.9	57.5 ± 0.49	3.2 ± 0.07	94.3	2.2 ± 0.03	0.7 ± 0.01	67.4
DGPC-59		20.3 ± 1.44	6.6 ± 0.45	67.3	30.9 ± 0.45	3.1 ± 0.03	89.7	1.6 ± 0.02	0.8 ± 0.04	50.1
Averag**e**		33.1	9.3*	71.7	43.7	5.6**	87.0	1.7	0.5*	67.4

Significant at *p = 0.05, **p = 0.01 and ***p = 0.001.

It was found that highest shoot length was observed in WBC-13 (20 cm), DARL-106 (15.3 cm) and lowest in DGPC-59 (6.6 cm), DC-206 (8.7cm) under stress conditions. Fresh weight was higher in WBC-13 (21.1 g), DGC-103 (15.5 g) and much lower low in DGPC-59 (3.1 g), DC-206 (3.2 g) under high temperature conditions. Highest dry weight was recorded in WBC-13 (2.6g) and WBC 39-1 (1.8 g) and lowest in WBC-22 (0.2 g) and EC-753493 (0.4g) under stress conditions. Significant effects were seen (p< 0.05) for the effects of genotype, temperature, and genotype × temperature interaction on shoot length, fresh weight, and dry weight (**Table 7**; **Table S4**).

**Table 7:** Evaluation of ten diverse genotypes for shoot length, fresh weight and dry weight under control, moderate and high temperature conditions.

		Control (m ± SE)	Treatment (m ± SE) (40°C/35°C)	Control (m ± SE)	Treatment (m ± SE) 40°C/35°C)	Control(m ± SE)	Treatment (m ± SE) 40°C/35°C)
Sl.no	Genotype	Shoot length		Fresh weight		Dry weight	
1	DARL106	21.6 ± 0.45^b^	15.3 ± 0.56^b^	40.0 ± 0.26^e^	12.9 ± 0.09^c^	2.3 ± 0.05^c^	1.3 ± 0.01^bcd^
2	DGC-103	23.2 ± 4.56^b^	14.1 ± 0.73^bc^	50.5 ± 0.17^c^	15.4 ± 0.22^b^	2.7 ± 0.01^b^	1.6 ± 0.02^bc^
3	WBC-13	28.0 ± 0.16^b^	20.0 ± 0.94^a^	45.4 ± 0.83^d^	21.1 ± 0.20^a^	4.1 ± 0.01^a^	2.6 ± 0.27^a^
4	WBC-39-1	23.3 ± 1.19^b^	14.6 ± 0.54^bc^	42.9 ± 0.42^d^	14.6 ± 0.18^b^	2.6 ± 0.05^b^	1.8 ± 0.13^b^
5	DC83	12.6 ± 0.76^b^	9.3 ± 0.49^de^	21.9 ± 0.12^h^	10.5 ± 0.05^d^	1.6 ± 0.02^d^	1.1 ± 0.04^cde^
6	BAROPATTA	52.7 ± 0.29^a^	11.2 ± 0.39^cd^	62.5 ± 0.40^a^	7.6 ± 0.10^e^	1.2 ± 0.02^e^	0.5 ± 0.01^ef^
7	EC-753493	26.3 ± 1.91^b^	10.1 ± 0.72^de^	32.1 ± 0.11^g^	6.5 ± 0.10^f^	1.3 ± 0.02^e^	0.4 ± 0.01^f^
8	WBC-22	17.5 ± 1.50^b^	10.0 ± 0.19^de^	35.5 ± 0.54^f^	7.8 ± 0.06^e^	2.3 ± 0.01^c^	0.2 ± 0.01^f^
9	DC-206	48.6 ± 8.31^a^	8.7 ± 0.53^de^	57.5 ± 0.49^b^	3.2 ± 0.07^g^	2.2 ± 0.03^c^	0.7 ± 0.01^def^
10	DGPC-59	20.3 ± 1.44^b^	6.6 ± 0.45^e^	30.9 ± 0.45^g^	3.1 ± 0.03^g^	1.6 ± 0.02^d^	0.8 ± 0.04^def^

Values within a group in a column bearing different letters are significantly different as determined by Tukey’s test.

### Biochemical traits

#### Proline content

Higher amount of proline accumulation was recorded in tolerant group of genotypes under heat stress conditions (**Supplementary Figure 5A**). Tolerant group accumulated 44.9% and 50.8% more proline in moderate temperature treatment (35°C/30°C) and high temperature treatment (40°C/35°C), respectively over control condition. There was no significant increase in proline level in susceptible group of genotypes under stress conditions (**Table 8**). The tolerant genotypes WBC-13 (15.93 µmol*g* ^-1^FW) and DC-83 (14.50 µmol*g* ^-1^FW) had accumulated maximum proline content whereas DC-206 (6.39 µmol*g* ^-1^FW) and EC-753493 (6.63 µmol*g* ^-1^FW) had minimum proline accumulation at 35°C/30°C treatment. Maximum proline was recorded in WBC-13 (17.9 µmol*g* ^-1^FW) and DARL-106 (15.0 µmol*g* ^-1^FW) and minimum accumulation was recorded in DC-206 (6.3 µmol*g* ^-1^FW) and EC-753493 (7.0 µmol*g* ^-1^FW) at 40°C/35°C treatment (**Table 9**). The effects of genotype, temperature, and genotype × temperature interaction for proline content were all significant (**Table S5**).

**Table 8:** Effect of high temperature treatment on biochemical parameters in cucumber genotypes under control, moderate and high temperature conditions.

Parameter	Tolerant group					Susceptible group				
	Control (mean)	35°C/30°C (mean)	Gain (%) at 35°C/30 °C	40°C/35 (mean)	Gain (%) at 40°C/35°C	Control(mean)	35°C/30°C (mean)	Gain (%) at 35°C/30°C	40°C/35°C (mean)	Gain (%) at 40°C/35°C
**Proline**	6.9	12.5**	44.9	14.0**	50.8	7.4	7.1ns	-4.1	7.2 ns	-2.6
**Super oxide dismutase**	26.5	34.5*	23.2	53.8*	50.7	31.7	16.7*	-56.5	33.8 ns	-6.5
**Catalase**	6.2	11.1**	44.4	12.8**	51.6	5.2	6.4*	19.8	6.8 ns	24.4
**Guaiacol peroxidase**	8.5	21.7**	60.7	22.8**	62.6	7.6	9.7*	21.8	10.4*	27.0
**Malondialdehyde**	11.5	16.6*	30.9	19.0**	39.6	12.1	24.5***	50.7	29.9***	129.3
**Protein**	1.8	3.4*	46.6	2.5*	29.2	1.8	1.1**	-65.9	0.8***	-112.0
**Ascorbate peroxidase**	5.1	5.4*	4.4	6.0*	13.7	4.4	3.6*	-26.7	2.7**	-62.4
**Hydrogen peroxidase**	25.6	20.2*	-29.2	14.5**	-84.1	22.0	25.9*	14.9	30.7**	27.8

Significant at *p = 0.05, **p = 0.01 and ***p = 0.001.

**Table 9:** Evaluation of ten diverse genotypes for proline, super oxide dismutase, catalase, guaiacol peroxidase, malondialdehyde, protein, ascorbate peroxidase and hydrogen peroxide under different temperature conditions.

		Control (m ± SE)		35°C/30°C (m ± SE)		Gain (%) at 35°C/30°C	40°C/35°C (m ± SE)	Gain (%) at 40°C/35°C	Control (m ± SE)	35°C/30°C (m ± SE)	Gain (%) at 35°C/30°C		40°C/35°C (m ± SE)	Gain (%) at 35°C/30°C
_Sl.no_	_Genotype_	_Proline_							_Super oxide dismutase_					
1	DARL106	6.4 ± 0.06^de^		12.1 ± 0.04^c^		46.5	15.0 ± 0.16^b^	56.9	17.6 ± 0.13^e^	26.2 ± 0.61^c^	32.7		43.9 ± 1.10^de^	59.9
2	DGC-103	7.0 ± 0.11^c^		10.3 ± 0.03^d^		31.8	12.9 ± 0.06^c^	45.2	29.0 ± 0.09^d^	33.7 ± 0.68^b^	13.9		39.2 ± 0.04^e^	26.1
3	WBC-13	6.3 ± 0.14^e^		15.9 ± 0.12^a^		60.2	17.9 ± 0.39^a^	64.7	18.5 ± 0.37^e^	34.4 ± 0.30^b^	46.0		72.8 ± 1.77^a^	74.5
4	WBC-39-1	6.6 ± 0.14^cde^		9.6 ± 0.13^e^		31.3	11.5 ± 0.19^c^	42.9	31.2 ± 0.13^c^	33.1 ± 0.43^b^	5.7		53.9 ± 1.32^c^	42.1
5	DC83	7.9 ± 0.15^b^		14.5 ± 0.12^b^		45.2	12.6 ± 0.30^c^	37.2	36.1 ± 0.36^b^	45.2 ± 0.12^a^	20.0		5 9.0 ± 0.89^b^	38.7
6	BAROPATTA	7.0 ± 0.09^cd^		7.7 ± 0.12^f^		9.6	7.0 ± 0.12^de^	2.1	11.2 ± 0.08^f^	21.6 ± 0.52^d^	48.0		41.2 ± 0.94^de^	72.7
7	EC-753493	7.8 ± 0.11^b^		6.6 ± 0.06^h^		-18.7	7.0 ± 0.36^de^	-11.6	35.1 ± 0.16^b^	16.8 ± 0.26^ef^	-109.4		26.3 ± 0.27^f^	-33.5
8	WBC-22	8.8 ± .03^a^		7.3 ± 0.06^fg^		-19.4	8.0 ± 0.18^d^	-9.2	17.0 ± 0.21^e^	10.7 ± 0.23^g^	-58.4		39.6 ± 0.14^e^	57.0
9	DC-206	6.3 ± 0.08^e^		6.3 ± 0.04^h^		0.8	6.3 ± 0.47^e^	0.0	31.2 ± 0.02^c^	18.7 ± 0.15^e^	-66.4		16.2 ± 0.37^g^	-91.6
10	DGPC-59	6.8 ± 0.11^cde^		7.2 ± 0.07^g^		5.8	7.2 ± 0.16^de^	6.5	63.7 ± 0.90^a^	15.4 ± 0.06^f^	-312.0		45.2 ± 1.13^d^	-40.9
Sl.no	Genotype	Catalase							Guaiacol Peroxidase					
1	DARL106	4.4 ± 0.03^e^		9.0 ± 0.21^c^		51.1	12.4 ± 0.29^b^	64.5	11.4 ± 0.17^a^	19.5 ± 0.41^c^	41.6		23.3 ± 0.07^b^	51.2
2	DGC-103	10.0 ± 0.07^a^		12.4 ± 0.14^b^		19.4	13.2 ± 0.34^b^	24.2	6.7 ± 0.08^cde^	22.4 ± 0.30^b^	69.8		23.2 ± 0.42^b^	70.9
3	WBC-13	5.2 ± 0.12^c^		13.4 ± 0.23^a^		61.2	15.0 ± 0.17^a^	65.3	11.8 ± 0.07^a^	22.0 ± 0.48^b^	46.2		17.1 ± 0.31^d^	30.8
4	WBC-39-1	6.8 ± 0.14^b^		9.2 ± 0.14^c^		26.1	10.8 ± 0.06^c^	37.0	6.0 ± 0.11^de^	18.8 ± 0.03^c^	68.1		19.8 ± 0.03^c^	69.8
5	DC83	4.6 ± 0.11^de^		11.8 ± 0.15^b^		61.0	12.6 ± 0.18^b^	63.5	6.5 ± 0.04^cde^	25.5 ± 0.21^a^	74.2		30.3 ± 0.38^a^	78.3
6	BAROPATTA	5.0 ± 0.06^cd^		7.0 ± 0.03^de^		28.6	8.6 ± 0.12^d^	41.9	5.3 ± 0.13^e^	7.7 ± 0.01^g^	30.8		9.3 ± 0.19^g^	42.0
7	EC-753493	4.4 ± 0.05^e^		6.8 ± 0.16^e^		35.3	6.8 ± 0.06^e^	35.3	8.9 ± 0.16^bc^	10.2 ± 0.16^e^	13.3		11.8 ± 0.15^ef^	24.4
8	WBC-22	6.8 ± 0.13^b^		7.6 ± 0.08^d^		10.5	7.2 ± 0.08^e^	5.6	6.0 ± 0.15^de^	8.7 ± 0.06^fg^	31.4		10.9 ± 0.27^f^	44.8
9	DC-206	5.0 ± 0.01^cd^		5.6 ± 0.07^f^		10.7	7.0 ± 0.07^e^	28.6	7.8 ± 0.02^bcd^	12.3 ± 0.21^d^	36.5		12.8 ± 0.03^e^	39.0
10	DGPC-59	4.8 ± 0.01^cde^		5.4 ± 0.21^f^		11.1	4.8 ± 0.03^f^	0.0	9.7 ± 0.05^ab^	9.3 ± 0.07^ef^	-4.7		7.1 ± 0.10^h^	-36.9
Sl.no	Genotype	Malondialdehyde							Protein					
1	DARL106	11.3 ± 0.11^de^		14.1 ± 0.29^fg^		20.0	17.2 ± 0.42^f^	34.3	2.1 ± 0.02^b^	3.1 ± 0.03^c^	31.8		1.9 ± 0.02^d^	-9.1
2	DGC-103	13.0 ± 0.30^b^		15.0 ± 0.27^f^		13.7	15.6 ± 0.01^g^	16.5	1.2 ± 0.02^e^	3.1 ± 0.01^c^	59.8		2.4 ± 0.05^c^	49.1
3	WBC-13	12.6 ± 0.26^bc^		17.6 ± 0.23^e^		28.5	20.3 ± 0.14^e^	38.0	2.4 ± 0.06^a^	3.8 ± 0.03^b^	36.8		3.0 ± 0.08^b^	20.0
4	WBC-39-1	7.3 ± 0.08^f^		13.6 ± 0.13^g^		46.2	16.5 ± 0.08^fg^	55.5	2.1 ± 0.01^b^	2.7 ± 0.02^d^	22.0		1.7 ± 0.01^e^	-18.5
5	DC83	13.1 ± 0.27^ab^		22.5 ± 0.24^cd^		41.7	25.4 ± 0.34^d^	48.2	1.0 ± 0.01^f^	4.0 ± 0.04^a^	73.6		3.4 ± 0.01^a^	69.0
6	BAROPATTA	10.3 ± 0.10^e^		21.4 ± 0.42^d^		51.8	30.4 ± 0.21^c^	66.1	1.6 ± 0.01^d^	0.6 ± 0.01^h^	-169.0		0.4 ± 0.01^h^	-270.7
7	EC-753493	14.1 ± 0.10^a^		25.0 ± 0.25^b^		43.3	32.9 ± 0.48^b^	56.9	1.9 ± 0.05^c^	1.6 ± 0.03^e^	-18.4		1.1 ± 0.02^f^	-77.7
8	WBC-22	13.1 ± 0.32^ab^		23.2 ± 0.07^c^		43.3	25.0 ± 0.33^d^	47.4	1.7 ± 0.02^d^	1.3 ± 0.02^f^	-33.8		0.8 ± 0.01^g^	-102.4
9	DC-206	11.0 ± 0.07^de^		24.6 ± 0.36^b^		55.0	26.1 ± 0.38^d^	57.6	1.6 ± 0.04^d^	0.9 ± 0.01^g^	-81.2		0.9 ± 0.01^g^	-80.1
10	DGPC-59	11.6 ± 0.24^cd^		28.2 ± 0.34^a^		58.9	34.9 ± 0.29^a^	66.8	1.9 ± 0.04^bc^	0.9 ± 0.02^g^	-113.7		0.9 ± 0.01^g^	-118.7
Sl.no	Genotype	Ascorbate Peroxidase							Hydrogen Peroxidase					
1	DARL106	5.4 ± 0.03^b^		5.9 ± 0.06^a^		8.7	6.5 ± 0.06 ^a^	17.0	29.8 ± 0.19 ^b^	27.4 ± 0.03 ^b^	-8.8		18.3 ± 0.11^e^	-63.1
2	DGC-103	4.8 ± 0.02^c^		5.0 ± 0.04^b^		3.1	5.6 ± 0.10 ^b^	14.7	19.2 ± 0.34^g^	12.4 ± 0.10 ^h^	-54.6		9.5 ± 0.02^h^	-102.1
3	WBC-13	5.8 ± 0.10^a^		6.0 ± 0.02^a^		3.9	6.5 ± 0.09 ^a^	10.5	23.9 ± 0.18^d^	21.5 ± 0.25 ^f^	-11.2		16.4 ± 0.18^f^	-45.5
4	WBC-39-1	4.9 ± 0.01^c^		5.0 ± 0.07^b^		0.2	5.0 ± 0.02 ^c^	1.6	21.4 ± 0.04^ef^	17.5 ± 0.22 ^g^	-22.2		16.3 ± 0.10^f^	-31.4
5	DC83	4.8 ± 0.0^c^		5.1 ± 0.08^b^		5.9	6.4 ± 0.11^a^	24.7	33.6 ± 0.07^a^	22.5 ± 0.38 ^ef^	-49.3		12.1 ± 0.13^g^	-178.4
6	BAROPATTA	4.9 ± 0.10 ^c^		4.5 ± 0.05^c^		-8.1	3.4 ± 0.01^d^	-42.3	26.1 ± 0.40 ^c^	30.4 ± 0.21^a^	14.1		35.6 ± 0.24^a^	26.7
7	EC-753493	4.4 ± 0.02 ^d^		4.2 ± 0.04^d^		-3.9	2.7 ± 0.05^e^	-61.3	22.4 ± 0.04 ^e^	25.1 ± 0.36 ^cd^	10.6		27.6 ± 0.54^d^	18.8
8	WBC-22	3.8 ± 0.07 ^e^		3.1 ± 0.04^e^		-21.9	2.2 ± 0.01^f^	-73.9	21.6 ± 0.05^ef^	23.7 ± 0.33 ^de^	9.0		27.0 ± 0.36^d^		20.0
9	DC-206	4.9 ± 0.03 ^c^		2.9 ± 0.01^e^		-67.2	2.7 ± 0.02^e^	-84.9	20.7 ± 0.01^f^	25.8 ± 0.23 ^c^	19.6		30.3 ± 0.09^c^	31.6
10	DGPC-59	4.0 ± 0.07 ^e^		3.0 ± 0.01^e^		-32.5	2.7 ± 0.02^e^	-49.5	19.3 ± 0.25^g^	24.5 ± 0.19 ^de^	21.3		33.1 ± 0.62^d^	41.8

Values within a group in a column bearing different letters are significantly different as determined by Tukey’s test.

#### Super oxide dismutase content

In our study, the activity of superoxide dismutase enhanced with variable magnitude under heat stress conditions. **Supplementary Figure 5B** shown that amount of SOD accumulated in three conditions and significance difference among genotypes were recorded both in control and high temperature treatments. **Table 8** indicated that SOD level increased by 23.2% and 50.7% under moderate and high heat stress conditions in tolerant group compared to control conditions, respectively. Among tolerant genotypes, maximum percent of SOD accumulated in DC-83 (45.2 U*g* ^-1^FW *min* ^-1^) and WBC-13 (34.3 U*g* ^-1^FW *min* ^-1^) whereas low SOD was observed in WBC-22 (10.7 U*g* ^-1^FW *min* ^-1^) and DGPC-59 (15.4 U*g* ^-1^FW *min* ^-1^) at 35°C/30°C treatment. In high temperature stress (40°C/35°C treatment), WBC-13 (72.8 U*g* ^-1^FW *min* ^-1^) accumulated highest SOD followed by DC-83 (59.0 U*g* ^-1^FW *min* ^-1^). Among the susceptible genotypes DC-206 (16.2 U*g* ^-1^FW *min* ^-1^) and EC-753493 (26.3 U*g* ^-1^FW *min* ^-1^) had accumulated lowest SOD content (**Table 9**). There was significant effects of genotype, temperature, and genotype × temperature interaction for SOD (**Table S5**).

#### Catalase content

Catalase content in all genotypes under control and heat treatments conditions are depicted in **Supplementary Figure 5C**. Catalase content has increased in all genotypes irrespective of tolerance and susceptibility, but tolerant genotypes accumulated higher amount of catalase compared to susceptible genotypes. Percent increase in catalase was 44.4% and 51.6% in tolerant genotypes where as in susceptible genotypes, percentage increase was by 19.8% and 24.4% in moderate and high temperature stress conditions, respectively (**Table 8**).

Highest catalase activity was recorded in WBC-13 (13.4 U*g* ^-1^FW *min* ^-1^) and DGC-103 (12.4 U*g* ^-1^FW *min* ^-1^) and lowest in DGPC-59 (5.4 U*g* ^-1^FW *min* ^-1^) and DC-206 (5.6 U*g* ^-1^FW *min* ^-1^) at 35°C/30°C treatment. The genotypes WBC-13(13.2 U*g* ^-1^FW *min* ^-1^) and DGC-103 (13.2 U*g* ^-1^FW *min* ^-1^) had recorded highest catalase activity and DGPC-59 (4.8 U*g* ^-1^FW *min* ^-1^), EC-753493 (6.8 U*g* ^-1^FW *min* ^-1^) had lowest catalase activity at 40°C/35°C treatment (**Table 9**). Temperature, genotype, and genotype × temperature interaction was significant for catalase (**Table S5**).

#### Guaiacol peroxidase content

Peroxidase content of leaves increased significantly with increase in temperature than that from control conditions in all genotypes (**Supplementary Figure 5D**). Significant differences were noted among genotypes under different conditions. In tolerant genotypes GPX increased by 60.7% and 62.6%, where as in susceptible genotypes amount increased by 21.8% and 27% in moderate and high temperature stress conditions, respectively over control conditions (**Table 8**). Maximum peroxidase activity was recorded in DC-83 (25.55 µmolg^-1^min^-1^) and DGC-103 (22.43 µmolg^-1^min^-1^) and minimum in Baropatta (7.78 µmolg^-1^min^-1^) and WBC-22 (8.78 µmolg^-1^min^-1^) at 35°C/30°C treatment. At 40°C/35°C treatment, lowest peroxidase activity was recorded in DGPC-59 (7.13 µmolg^-1^min^-1^), Baropatta (9.30 µmolg^-1^min^-1^) and highest was in DC-83 (30.36 µmolg^-1^min^-1^), DARL-106 (23.28 µmolg^-1^min^-1^) at high temperature stress conditions (**Table 9**). There were significant (p<0.05) effects of genotype, temperature, and genotype × temperature interaction on GPX content (**Table 5S**).

#### Malondialdehyde content

In response of high temperature stress, high endogenous malondialdehyde content levels were observed in cucumber plants (**Supplementary Figure 5E**). In susceptible genotypes, malondialdehyde content significantly increased with increase in temperature as compared to control conditions. Malondialdehyde content increased by 50.7% and 129.3% in susceptible group in contrast to tolerant group with an increase by 30.9% and 39.6% in moderate and high temperature stress conditions, respectively. (**Table 8**). The genotype DGPC-59 (28.25 nmolg^-1^FW, 34.9 nmolg^-1^FW) had shown highest malonaldehyde content under both high temperature treatments whereas WBC 39-1 (13.6 nmolg^-1^FW) and DGC-103(15.6 nmolg^-1^FW) had shown lowest malonaldehyde content in 35°C/30°C treatment and 40°C/35°C treatment, respectively (**Table 9**). Temperature, genotype, and genotype × temperature interaction all had significant effects on Malondialdehyde accumulation in the plants (**Table S5**).

#### Protein content

Greater increase in protein content was noticed in 35°C/30°C treatment compared to 40°C/35°C treatment (**Supplementary Figure 5F**). Tolerant group accumulated 46.6% and 29.2% of protein in moderate and high temperature conditions over the control, whereas protein level was significantly reduced in susceptible genotypes (**Table 8**). Highest protein was recorded in DC-83 (4.05 mg g^-1^, 3.45 mg g^-1^) followed by WBC-13 (3.8 mg g^-1^, 3.03 mg g^-1^) and lowest was recorded in Baropatta (0.6 mg g^-1^, 0.4 mg g^-1^) and DC-206 (0.9 mg g^-1^, 0.9 mg g^-1^) under both treatment conditions (**Table 9**). Significant difference has been observed among genotypes under all three conditions. The effects of genotype, temperature, and genotype × temperature interaction on protein content were significant (**Table S4**).

#### Ascorbate peroxidase content

Greater increase in ascorbate peroxidase was noticed 40°C/35°C treatment in treatment compared to 35°C/30°C (**Supplementary Figure 5G**). Tolerant group accumulated 4.4% and 37.6% of APX content in moderate and high temperature conditions over the control, whereas APX level was significantly reduced in susceptible genotypes (**Table 8**). Highest APX was recorded in WBC-13 (6.0 µmolmin^-1^g^-1^1 6.5 µmolmin^-1^g^-1^) in moderate and high stress conditions. Lowest was recorded in DC-206 (2.9 µmolmin^-1^g^-1^) under moderate stress and WBC 22 (2.2 µmolmin^-1^g^-1^) under high stress conditions (**Table 9**). The effects of genotype, temperature, and genotype × temperature interaction on protein content were significant (**Table S4**).

#### Hydrogen peroxidase content

In response of high temperature stress, high levels of hydrogen peroxide was content levels were observed in cucumber plants (**Supplementary Figure 5H**). In susceptible genotypes, hydrogen peroxide content significantly increased with increase in temperature as compared to control conditions. Hydrogen peroxidase content increased by 14.9% and 27.8% in susceptible group in contrast to significant decrease in tolerant at stress conditions (**Table 8**). The genotype Baropatta(30.4 µmolg^-1^FW, 35.6 µmolg^-1^FW) had shown highest hydrogen peroxidase content under both high temperature treatments whereas DGC-103(12.4 µmolg^-1^FW, 9.5 µmolg^-1^FW) had shown lowest hydrogen peroxidase content in moderate and high stress conditions (**Table 9**). The effects of genotype, temperature, and genotype × temperature interaction on hydrogen peroxidase content were significant (**Table S4**).

### RT-PCR of selected genes associated with heat stress

**Figure 3:**
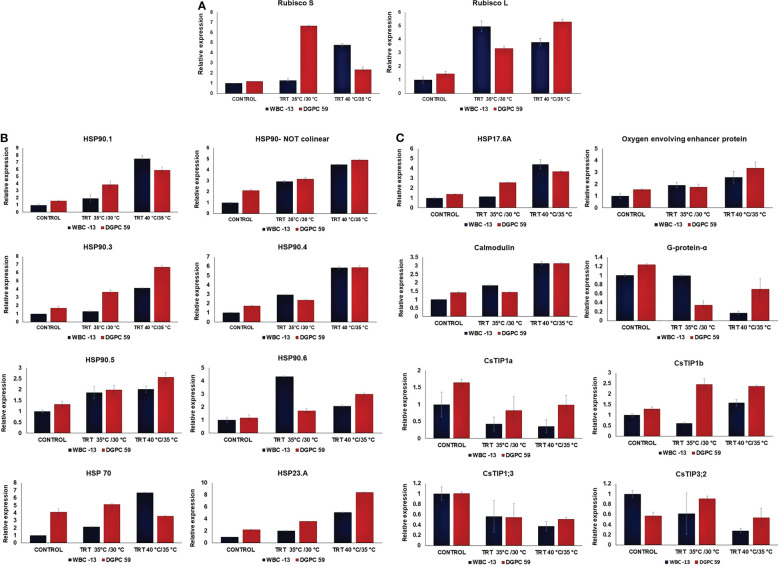
**(A)** Relative expression (means± SE) of Photosynthesis related genes (Rubisco S and Rubisco L) in cucumber leaves under in control, moderate temperature treatment (35°C/30°C) and high temperature treatment (40°C/35°C). **(B)** Relative expression (means± SE) of Heat shock proteins (HSPs) in cucumber leaves under in control, moderate temperature treatment (35°C/30°C) and high temperature treatment (40°C/35°C). **(C)** Relative expression (means± SE) of HSP 17.6 A and signal transduction genes in cucumber leaves under in control, moderate temperature treatment (35°C/30°C) and high temperature treatment (40°C/35°C).

Based on the performance and analysis of physio-biochemical characters in 10 different genotypes, two genotypes namely, WBC-13 (heat tolerant) and DGPC-59 (heat susceptible) were selected for gene expression studies of selected heat responsive genes. Expression profiling of 18 selected heat responsive genes was conducted in two contrasting genotypes under three different temperature conditions (Control: 30°C/25°C, Moderate stress: 35°C/30°C and High stress: 40°C/35°C). Genes used in study are mentioned in **Table S5**. Rubisco S gene showed highest expression of 6-7 folds at moderate heat stress in DGPC-59 whereas WBC-13 showed highest expression of 4-5 folds at high stress condition. Analysis of result showed that Rubisco L gene was 5-6 folds upregulated in DGPC-59 at high heat stress whereas WBC-13 showed increase in 5-6 folds expression at moderate heat stress (**Figure 3A**).

A remarkable up-regulation in relative accumulation of some HSPs under high stress conditions were recorded in tolerant genotypes. At the same time, few HSPs were shown down regulation under high heat stress conditions in the tolerant genotype, WBC-13 (**Figure 3B**). In control conditions, no significant differences were observed between the genotypes except for the HSP70 which showed higher expression in susceptible genotypes. It was observed that at moderate heat stress condition, only HSP 90.6 shown higher expression in WBC-13 as compared with other stress condition. At high heat stress condition, HSP 90.1, HSP 70 and HSP 17.6A shown higher expression in tolerant genotype, WBC-13. Some HSPs like HSP90.3, HSP90.6, HSP 23.A, HSP 90.5 shown upregulation in DGPC-59 whereas HSP90.4 shown no significant difference between the genotypes at high heat stress condition. Additionally, the genes corresponding to signal transduction were also studied under control and heat stress conditions. In control and treatment conditions, significant differences were seen among the genotypes (**Figure 3C**). Under high heat stress conditions tolerant genotype, WBC-13 expressed downregulation for CsTIP1;3, CsTIP3;2, CsTIP1a whereas WBC-13 shown upregulation for CsTIP1b gene (**Figure 5**). In case of Calmodulin gene expression, both contrasting genotypes had same level of expression with no significant difference. G-protein-α and oxygen evolving enhancer genes were upregulated in DGPC-59 at high heat stress condition and downregulated in WBC-13.

### Correlation analysis

#### Moderate temperature stress (35°C/30°C)

**Figure 4:**
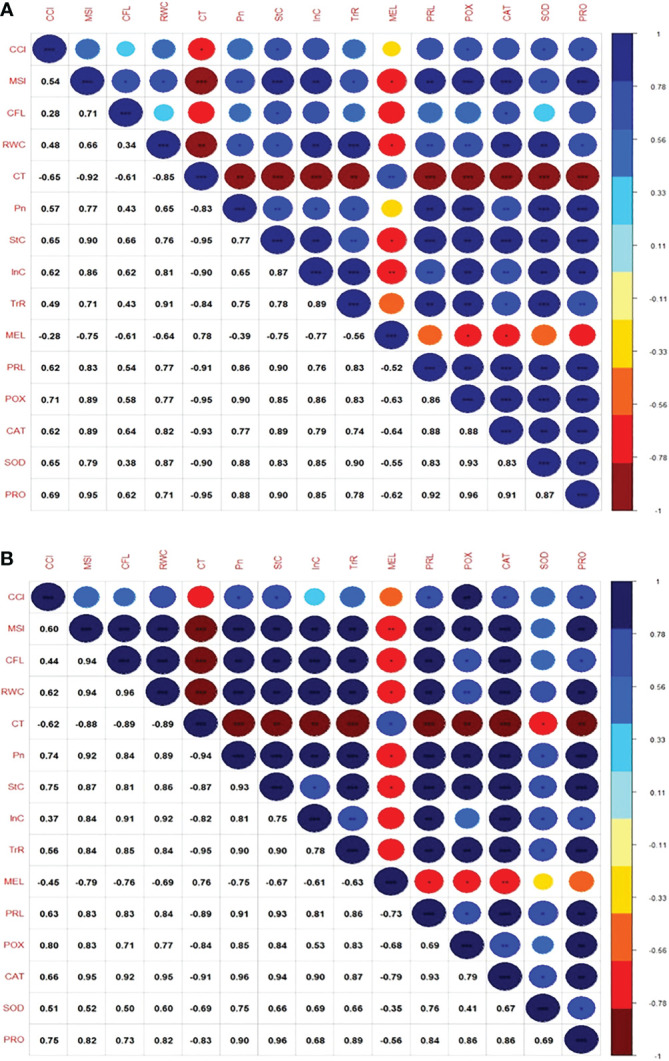
**(A)** Correlogram showing the relationship between average values of the variables at moderate high temperature stress conditions 35°C/30°C. **(B)** Correlogram showing the relationship between average values of the variables at moderate high temperature stress conditions 40°C/35°C; The intensity of color and size of the circle increases with an increase in the significance of correlation. Dark red denotes a high negative correlation, whereas dark blue denotes a high positive correlation.

A correlogram (**Figure 4A**) depicts the correlation between the all parameters measured between all parameters in all genotypes at moderate heat stress conditions 35°C/30°C. Chlorophyll content (CCI) was significantly and positively correlated with membrane stability index (r=0.54), relative water content (r=0.48), net photosynthesis (r=0.57) and transpiration rate(r= 0.49), proline (r=0.62), catalase (r=0.62), stomatal conductance (r=0.65), internal CO_2_ concentration (r=0.62), peroxidase (r=0.71), super oxide dismutase (r=0.65), protein (r=0.69) and with chlorophyll florescence (r=0.33). However, there was a significant negative correlation of the parameters chlorophyll and canopy temperature (r=-0.78) and malonaldehyde (r=-0.33). The MSI was positively and significantly correlated to stomatal conductance (r=0.90), internal CO_2_ concentration (r=0.86), proline(r=0.83), peroxidase(r=0.89), catalase (r=0.89), protein (r=0.95), chlorophyll florescence(r=0.71), relative water content(r=0.66), net photosynthesis(r=0.77), transpiration rate (r=0.71) and super oxide dismutase (0.79) and there was a significant negative correlation of the parameters MSI and CT (r=-0.1) and malonaldehyde (r=-0.78). Chlorophyll fluorescence is positively correlated with stomatal conductance, catalase, internal CO_2_ concentration, protein with r value 0.78 and net photosynthesis, transpiration rate, proline, peroxidase with r=0.56 and relative water content and super oxide dismutase with r=0.33, negative correlation exists between CFC and malonaldehyde and canopy temperature with r=-0.78. Similarly, canopy temperature was positively correlated with malonaldehyde (r=0.78). Additionally, net photosynthesis is positively correlated with proline, peroxidase, SOD, and protein with r value near to 1 and with stomatal conductance, internal CO_2_ concentration, transpiration rate and catalase with r=0.78. Besides, internal CO_2_ concentration was positively correlated with transpiration rate, peroxidase, SOD and proline content. In edition biochemical parameters proline, peroxidase, SOD and protein are positively and significantly correlated to each other.

#### High temperature stress (40°C/35°C)

A correlogram (**Figure 4B**) depicts the correlation between the all parameters measured between all parameters in all genotypes at moderate heat stress conditions 40°C/35°C. Chlorophyll content (CCI) is significantly and positively correlated with peroxidase (r=0.8), relative water content(r=0.62), membrane stability index (r=0.60), net photosynthesis (r=0.74), stomatal conductance (r=0.75), proline (r=0.74), catalase (r=0.66) and protein (r=0.75), chlorophyll florescence (0.44), transpiration rate (r=0.56), super oxide dismutase (r=0.51), internal CO_2_ concentration (r=0.33) and CCI is negatively correlated with canopy temperature (r = -0.78), malonaldehyde (r=-0.56). Membrane stability index was highly correlated with chlorophyll florescence, relative water content, net photosynthesis, stomatal conductance, internal CO_2_ concentration, transpiration rate, proline, peroxidase, catalase and protein with r value near to 1. SOD (r=0.56) and MSI were negatively correlated with malonaldehyde (r=-0.78) and canopy temperature (r=-1). Chlorophyll fluorescence had significant positive correlation with relative water content, net photosynthesis, stomatal conductance, internal CO_2_ concentration, transpiration rate, proline, catalase with r value near to 1 and peroxidase and protein with r value near to 0.78, SOD (r=0.56) and negative correlation with malonaldehyde (r=-0.78) and canopy temperature (r=-1). Relative water content had significant positive correlation (r=1) with parameters net photosynthesis, stomatal conductance, internal CO_2_ concentration, transpiration rate, proline and catalase and protein and peroxidase (r=0.78), SOD (r=0.56) and negatively correlated with Malondialdehyde (-0.78) and canopy temperature (r=-1). Canopy temperature shown positive significance with malondialdehyde (r=0.78). Net photosynthesis was significantly and positively correlated with stomatal conductance, internal CO_2_ concentration, transpiration rate, proline, peroxidase, catalase and protein with r value near to 1, SOD with r=0.78 and negatively correlated with malondialdehyde content (-0.78). Stomatal conductance and transpiration rate were showing significant and positive correlation with proline, peroxidase, catalase, SOD, protein except with malondialdehyde. Biochemical parameters had positive correlation among themselves except malondialdehyde content.

## Discussion

**Figure 5:**
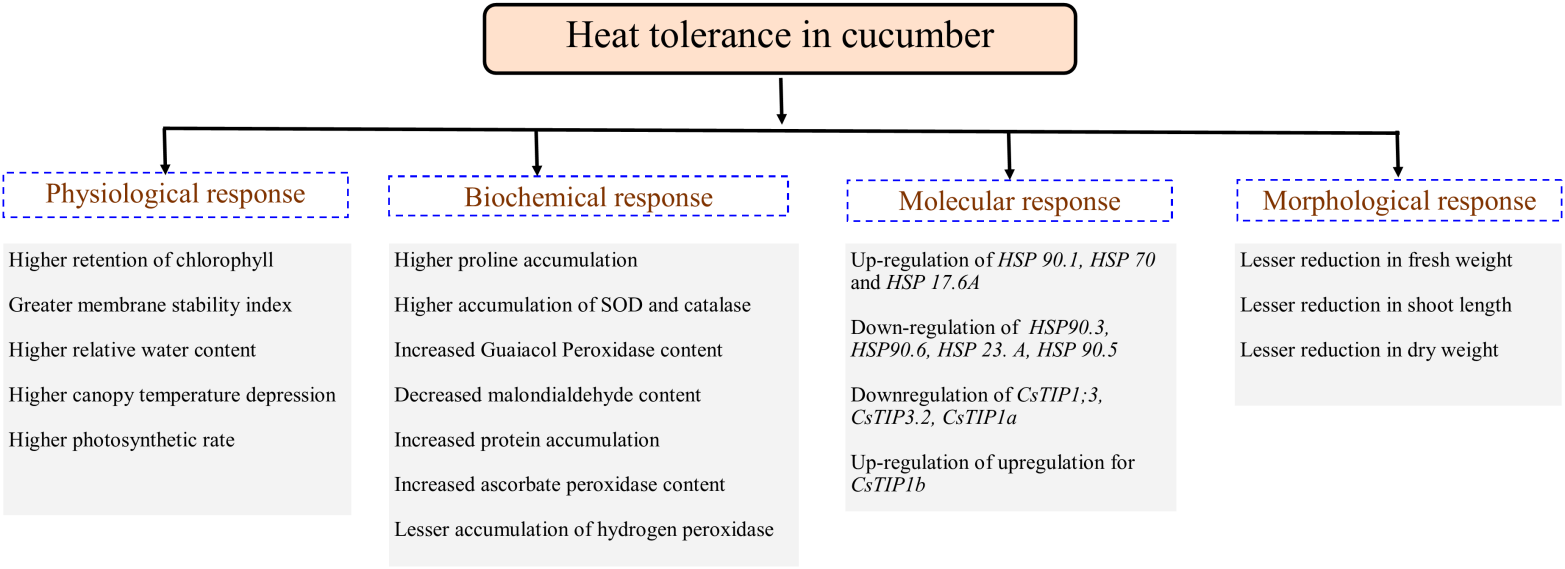
Important physiological, biochemical, molecular and morphological factors associated with heat stress tolerance in cucumber.

Plants being sessile are constantly exposed to several abiotic stresses comprising drought, heat or different stress combinations that results in several metabolic disparities leading to oxidative damage due to ROS production and accumulation. ROS build-up in plants triggers organelle integrity, oxidation of cellular components, and even can lead to cell death (Suzuki et al., 2014; Nath et al., 2016; Raja et al., 2017). Thus, plants may have evolved different physiological, biochemical and molecular mechanisms to adopt for heat stress conditions. We are reporting the comprehensive physio-biochemical response of a contrasting set of cucumber genotypes under varied level of high-temperature stress conditions (**Figure 5**). Expression analysis of selected important genes were performed to examine the molecular responses of the cucumber genotypes to heat stress.

### Physiological basis of heat stress tolerance

In our present experiment, analysis of variance for different physiological and biochemical parameters was significant indicating the existence of significant genetic variability among the genotypes and differential response of genotypes to heat stress conditions. In chloroplast, the chlorophyll is harbored by thylakoid which are considered as the most heat labile cell structures (Vacha et al., 2007). Any damage to thylakoid caused by heat is expected to result in chlorophyll loss. This showed that chlorophyll is linked with dry matter accumulation and can be utilized in screening the genotypes for high-temperature tolerance at seedling stage of cucumber. We found variations in the percent degradation of chlorophyll in the present investigation suggesting that some of the cucumber genotypes were able to maintain more chlorophyll under stress conditions. Chlorophyll content in leaves decreased as HT days increased, and the decrease was faster in a heat-susceptible cultivar compared to tolerant cultivar as was observed in other crops like hot pepper (Arnaoudova et al., 2020) and tomato genotypes (Bhattarai et al., 2021). The higher chlorophyll content in heat-tolerant cultivar gives better photosynthetic stability than heat-susceptible tomato cultivars (Zhou et al., 2017). By elevating unsaturated fatty acids and making the plasma membrane more fluid, HS causes the plasma membrane to become disorganized (Hofmann, 2009). It also affects cellular processes by starting a signal cascade (Firmansyah and Argosubekti, 2020; Hassan et al., 2021). The ability to adapt to high temperatures appears to be governed by a stable cell membrane system that keeps working under heat stress. High temperature stress can directly affect membrane integrity through photochemical modifications during photosynthesis or ROS (Bita and Gerats, 2013). It is also suggested that the membrane disruption may alter water, ion and organic solutes movement across the plant membrane which interferes with photosynthesis and transpiration. In this study we evaluated the membrane stability index and it was found that tolerant genotypes maintained high stability index. Recently, it was demonstrated that tolerant genotype of cucumber shown less relative conductivity and less damage of membrane lipid (Wang et al., 2020). The thermosensitive cucumber genotypes had high relative conductivity after high-temperature stress compared to tolerant genotypes (Yu et al., 2022). Similarly, cucumber genotypes with less electrolyte leakage ability tend to be more heat tolerant than genotypes with more electrolyte leakage at a high temperature of 40°C (Ali et al., 2019). Heat-tolerant cultivars of cauliflower expressed more cell membrane thermostability than susceptible ones (Aleem et al., 2021). Under different stress conditions, plants maintain their physiological balance through higher RWC values particularly under higher rates of transpiration. In the present study, plants subjected to heat stress conditions displayed decreased RWC values suggesting the sensitivity of cucumber plants towards heat stress. The difference in RWC under stress conditions did not differ significantly among the tolerant and susceptible genotypes suggesting its limited role in heat tolerance in cucumber. Raja et al. (2020) reported decreased RWC in tomato plants under high-temperature stress.

Chlorophyll fluorescence is a rapid, reliable, and inexpensive procedure for predicting photosynthetic performance under HS. Reduced *Fv/Fm* values indicate damage to the light-harvesting complex (Moradpour et al., 2021). Chlorophyll fluorescence has been used as screening tool in common bean (Stefanov et al., 2011) and okra (Hayamanesh, 2018). Several recent studies also supported our finding that tolerant genotypes shown higher *Fv/Fm* ratio with stable photosynthetic system under heat stress conditions. High temperature seriously impaired the photosynthetic system of susceptible plants as compared to the tolerant genotypes (Yu et al., 2022).

In our study, HT significantly decreased the photosynthetic rate in thermos-sensitive genotypes but stable rate of photosynthesis was maintained in the tolerant cultivar. The stable photosynthetic rate of heat-tolerant cultivar in HT might be due to the increased stomatal conductivity and transpiration rate. Under the condition of 40°C/35°C heat treatment, rate of photosynthesis and stomatal conductance were drastically reduced in the susceptible genotypes in contrast to the tolerant genotypes suggesting the ability of the tolerant genotypes to sustain the photosynthetic activities even under high-temperature stress conditions. Yu et al. (2022) demonstrated thermo-tolerant plants showed stable photosynthesis under high-temperature stress whereas sensitive plants had extremely unstable photosynthesis in cucumber. Photosynthetic rate was significantly reduced in susceptible cultivar but not in tolerant seedlings even with the exposure to 42°C in hot pepper. Similarly, stomatal conductivity and transpiration rate was significantly higher in tolerant genotypes compared to susceptible genotypes (Rajametov et al., 2021). In tomato, high stomatal conductivity and transpiration rate under heat stress facilitate reduced canopy temperature in heat-tolerant genotypes, providing better protection for chlorophyll and maintaining a relatively high photosynthetic rate (Zhou et al., 2017). Therefore, it was concluded that the ability of the tolerant genotypes to maintain a stable photosynthetic rate was one of the important factors for thermotolerance.

### Growth parameters in response to heat stress

In the present study, plants were subjected to high temperature stress conditions exhibited reduction in shoot length, fresh weight and dry weight values suggesting the effect of heat stress on biomass of the plants. However, the tolerant genotypes were able to maintain the higher biomass compared to susceptible genotypes. Reduction in plant growth under high temperatures varied among the genotypes and tolerant genotypes exhibited significantly better morphological traits when compared with the sensitive genotypes in tomato (Shaheen et al., 2016). Lower height reduction under heat-stress in tolerant genotypes signified that their ability to maintain their growth properly when exposed to heat stress conditions. The changes in plant diameter under heat stress may be related to changes in stem tissue hydration. Reduction in growth because of reduced water content in cell and cell size is common when plants are exposed to high temperature stress conditions (Ashraf and Hafeez, 2004; Rodríguez et al., 2005). Besides, retarded relative growth in the susceptible genotypes because of reduction in net assimilation rate (NAR) was reported in maize, millet (Wahid et al., 2007) and sugarcane (Srivastava et al., 2012).

### Biochemical basis of heat stress tolerance

Variability in increasing the activities of these antioxidants across cucumber genotypes indicates their differential ability to acquire thermo-tolerance. Even the heat tolerance was found directly linked with the percent increase in catalase, superoxide dismutase, guaiacol peroxidase accumulation in most genotypes. Thus, our results show that tolerance mechanism for heat stress exists in cucumber genotypes for a variable extent. Proline serves as a membrane protectant, and due to its zwitter ion character, accumulates in high-concentration in cell cytoplasm under stress conditions without interfering with cellular structure or metabolism. Proline in plants functions as an osmoprotectant and allows them to tolerate stress (Akram et al., 2018; Alzahrani et al., 2018). Higher levels of proline accumulation in plants occur under stress conditions. In this study, we evaluated the contents of proline and found that tolerant genotypes accumulated significantly higher proline content under heat stress in comparison to susceptible genotypes. Tomato genotypes accumulated high amount of proline under heat stress conditions (Raja et al., 2020). Proline content was also significantly higher in tolerant genotypes compared to susceptible genotypes after heat treatment in hot pepper (Rajametov et al., 2021). Several recent studies reported that proline accumulation occurs in plants with exposure to stress conditions (Kaur and Asthir, 2015; Moreno-Galván et al., 2020) because of its property to stabilize subcellular structures, scavenging free radicals and buffer cellular redox potential (Hazman et al., 2015; Dar et al., 2016; Nurdiani et al., 2018). Heat stress is known to accompany with the formation of reactive oxygen species such as H_2_O_2_ and OH^-^, which damage membranes and macromolecules. SOD is usually considered as the first line of defense against oxidative stress. In plants, we found significant difference between genotypes with respect to SOD accumulation and SOD activity was increased under heat stress conditions in tolerant genotype. No significant difference in superoxide dismutase enzyme (SOD) activity was detected between contrasting genotypes under normal conditions, whereas tolerant genotypes exhibited significant increase in SOD activity during heat stress compared with susceptible genotype (Wang et al., 2020). Tolerant genotypes of brinjal were reported to have higher amount of superoxide dismutase, peroxidase and catalase (Faiz et al., 2020). Superoxide dismutase (SOD) and peroxidase (POD) are two necessary antioxidant enzymes that protect plants from heat-induced oxidative stress. Catalase and peroxidises are the most important enzymes involved in regulation of intracellular level of H_2_O_2_. They convert H_2_O_2_ into OH^-^ along with the regeneration of NADP, thus helping the plants under stress conditions (Sairam et al., 1997; Xu et al., 2008). We found higher accumulation of catalase and peroxidises in tolerant genotypes under heat stress conditions compared to susceptible genotypes. The heat tolerance was found directly linked with the percent increase in catalase/superoxide dismutase/guaiacol peroxidase accumulation in wheat genotypes. During present study, MDA content was increased in susceptible genotypes as compared to tolerant genotypes under heat stress conditions. Potential resistance mechanisms of plants exposed to heat stress may involve higher osmotic regulation capacity related to an increase in leaf protein content (Ding et al., 2016). We also observed increased protein levels in tolerant genotypes under heat stress conditions was instrumental in conferring heat tolerance in cucumber genotypes.

### Gene expression in response to high temperature stress treatment

The acquisition of plant heat tolerance is closely associated with the synthesis of chaperone proteins and the levels of non-enzymatic antioxidants in response to HT (Kotak et al., 2007; Wahid et al., 2007). Heat shock proteins play an essential role in the regulation of HSFs and subsequently, the expression of heat responsive genes associated with heat tolerance. The plants generally activate and accumulate a large amount of the HSPs to response to heat shock exposure to maintain the stability of cells under stress conditions (Richter et al., 2010; Li et al., 2018). In our study, HSP 90.1 and HSP 70 shown seven-fold and six-fold increased expression, respectively in tolerant genotype in response to heat stress conditions. Earlier, Yu et al. (2018) also reported the role of heat shock proteins in thermotolerance in cucumber. Besides, lower expression of HSP70 has been recorded in susceptible genotypes of chilies in response to heat stress while the tolerant ones showed overexpression of HSP70 which enhanced the thermostability of cell membranes (Usman et al., 2015). Similarly, significant increase in BoHSP70 in cabbage, HSP60/CPN60, HSP70, HSP90, HSP100/ClpB, and HSP90 activator and HSP70/HSP90 organizing protein in spinach and ClHSP11.1A, ClHSP50.3, and ClHSP17.4 in watermelon are reported to play key role in response to high temperature stress (Park et al., 2013; He et al., 2018; Zhao et al., 2018). Recent studies revealed that chilli specific many HSPs including CaHSP70, CaHSP60, CaHSP20, and CaHSP16.4 are upregulated in pepper under HS and significant difference between the genotypes **(** Guo et al., 2015; Usman et al., 2015).

The AQPs channel proteins to facilitate the transport of water primarily through the plasma and tonoplast membranes in the plant cells (Chaumont and Tyerman, 2014). They are often designated as plasma membrane intrinsic proteins (PIPs) or tonoplast intrinsic proteins (TIPs) (Danielson and Johanson, 2008). Participation of AQPs in different abiotic stress responses is reported earlier (Matsumoto et al., 2009; Sreedharan et al., 2013; Wang et al., 2017). Besides, the differential expression of aquaporin isoforms is reported under different stress conditions (Alexandersson et al., 2010). Regulation of TIP controlled water transport across vacuolar membranes by different environmental signals is determined both at the transcriptional and the post-transcriptional levels (Li et al., 2014). Salinity, drought, gibberellic acid, and abscisic acid level are associated with regulation of TIPs (Hachez et al., 2006, Maurel et al., 2008; Moshelion et al., 2009; Hachez et al., 2012; Zarrouk et al., 2016). Transgenic approaches demonstrate that the overexpression of aquaporins in plants confers enhanced tolerance against abiotic stress, including drought (An et al., 2018; Lu et al., 2018; Wang et al., 2018; Patankar et al., 2019; Rosales et al., 2019; Wang et al., 2019). In the present study, varied expression of cucumber specific *CsAQPs* was recorded in the genotypes with contrasting response to heat stress. In control conditions, significant difference was found for genes *CsTIP3.2, CsTIP1a* in contrast to high temperature stress conditions. Therefore, *CsTIP3.2, CsTIP1a* were instrumental in providing heat stress response in the cucumber genotypes. No significant difference in calmodulin genes in the contrasting genotypes under the heat stress conditions indicated its limited role in conferring heat tolerance in cucumber. Upregulation of the G-protein-α and oxygen evolving enhancer genes in the susceptible genotype, DGPC-59 at high heat stress condition indicated their role as negative feedback in heat tolerance in cucumber. In pea and Chinese pear, induced level of the in response to the heat stress has been reported (Misra et al., 2007; Bhardwaj et al., 2020). Negative role of the GPA1 was also reported in *Arabidopsis* mutants in response to the heat stress (Chakraborty et al., 2015). Inhibition of electron transport from the oxygen evolving complex (OEC) of PSII is because of its dissociation by heat stress (Havaux and Tardy, 1996; Allakhverdiev et al., 1997; Wahid et al., 2007; Allakhverdiev et al., 2008). Therefore, negative feedback of the G-protein and OEC is established in cucumber in relation to the heat stress tolerance.

## Conclusion

Efforts to sustain crop production under steadily increasing global temperature remain imperative for food security. Exploring tolerance mechanisms to determine genotypes that can perform best under temperature extremes is of high priority to avoid significant shortage in food production in the following years. Thus, this study was conducted to explore the potential of different cucumber genotypes to sustain under high-temperature conditions and understanding the physio-biochemical and molecular mechanisms of high temperature tolerance in cucumber.

In our experiment, we categorized selected genotypes into various classes on the basis of important parameters analyzed in this study. Cucumber genotypes WBC-13 and DC-83 have been identified as high heat tolerant, and DGPC-59 and WBC-22 as highly heat susceptible whereas DARL-106, DGC-103, WBC-39-1 as tolerant and Baropatta, EC-753493, DC-206 as susceptible genotypes. We understood high chlorophyll retention, stable membrane stability index, higher retention of water content in plants, stability in net photosynthesis, good stomatal conductance and transpiration rate and maintaining less canopy temperatures in tolerant genotypes are the key physiological mechanisms in cucumber associated with heat tolerance. Accumulation of biochemicals like proline, protein and antioxidants like SOD, catalase and peroxidase formed the biochemical basis of high temperature tolerance. Upregulation of photosynthesis related genes, signal transduction genes and heat responsive genes (HSPs) in tolerant genotypes indicated their key role in determining molecular basis of heat stress tolerance in cucumber. These results indicate that the thermotolerant cucumber genotypes enhanced physio-biochemical and molecular adaptation under high-temperature stress conditions. It is suggested that these heat tolerant genotypes can be used in breeding programme, and information generated can be utilized in functional genomics in identifying the genomic regions and candidate genes associated with heat tolerance in cucumber.

## Data availability statement

The original contributions presented in the study are included in the article/**Supplementary Material**. Further inquiries can be directed to the corresponding authors.

## Author contributions

Conceived theme of the study and designed experiment: SD; Data curation: DH, DR, and SK; Investigation: DH, AD, SD, and KK; Resources: SD, TB, and AM, and VC; Supervision: SD, AM, TB; VC, AT, and PD; Visualization: SD, TB, and AM; Writing original draft: DH and SD; Review and editing: SD, TB, and AM. All authors contributed to the article and approved the submitted version.
