# Supplementary material for: Elucidating the role of key physio-biochemical traits and molecular network conferring heat stress tolerance in cucumber
Source: Front Plant Sci. 2023 Feb 20;14:1128928. doi: 10.3389/fpls.2023.1128928 (PMC9990136; doi:10.3389/fpls.2023.1128928)
Supplement: Supplementary file 1 [file DataSheet_1.docx]

**Table S1 :** Primers corresponding to genes used for expression analysis through RT-PCR under heat stress

| S.No | Gene name | Primer sequence ( 5’ -3’) | Primer sequence ( 3’ -5’) |
| --- | --- | --- | --- |
| 1 | Rubisco L | AGCCTGTTGCTGGAGAAG | AGGGCGACCATACTTGTT |
| 2 | Rubisco S | GCCTCAAATCTTCCGCTGGT | AATCCGCTTCCGATGTCGAAT |
| 3 | HSP90.1 | ACGAGTGGTCGTTGGTGAAC | AGAGAAGTGCTTTACGGCCA |
| 4 | HSP90- NOT colinear | CAAGAAGAAGGTTGAGAAGGTTG | CATGCTGTTGTCCTTCAATGCT |
| 5 | HSP90.3 | GTCCCAAAGAGGTCACGGAG | GGAGGCACAAACAGAACAGC |
| 6 | HSP90.4 | CTCTCCTTGCTGCTTGGTCA | TGTAAGCGCCCATGCTGTTA |
| 7 | HSP90.5 | CGGTGGTACTCGCTTGAGTT | CGCCTCACACCTAACAGCAA |
| 8 | HSP90.6 | TTCGCTCTGGTGGAGCTTAC | CACCGAATGGGAGAGGTGAG |
| 9 | HSP 70 | AGTGTTGTACCGCTAGTTGGA | GTGACCGAATCAACAGGTGC |
| 10 | Oxygen evolving enhancer protein | TCCTCCATCAATGGTTGGGC | TGTATTTCCAGGGAGCTGGT |
| 11 | Calmodulin | GAAGTGCATGAAGATCGCCA | TTTTCCGGTTTCAGATCGCG |
| 12 | HSP23.A | AAAACGAAGAAGATGGCGGC | ACATCGTTGCGTTCCTGTTC |
| 13 | HSP17.6A | GATCTGGAATTTGGCCGCTT | ATCCACCGCAAACACATACG |
| 14 | G-protein-α | ACCGAATGATGGAGACGAAGGAA | ACCATTCACACACACTGAGAGGGA |
| 15 | CsTIP1a | CATCGCAATCGGTTTCAT | AGTAGATCCAGTGGCTTTCC |
| 16 | CsTIP1b | TCGGCGGTAACATCACTAT | TTGGCAAGAATCCCGTAA |
| 17 | CsTIP1;3 | TGAATCCGGCAGTTACAT | CAACAACAGAGCCAAGCA |
| 18 | CsTIP3;2 | TTTAGTGGGCTGGAGATG | GGGCTGGTGAGTATGTAGAG |

**Table S2** ANOVA for important physiological parameters in contrasting set of cucumber genotypes

| **Source of variation** | **Df** | **Sum Sq** | **Mean Sq** | **F value** |
| --- | --- | --- | --- | --- |
| **Chlorophyll content** | | | | |
| **Genotype** | **9** | **614.7** | **68.3***** | **28.7** |
| **Temperature** | **2** | **435.8** | **435.8***** | **183.4** |
| **Genotype*temperature** | **18** | **109.2** | **12.1***** | **5.1** |
| **Residuals** | **70** | **166.2** | **2.4** |  |
| **Membrane stability index** | | | | |
| **Genotype** | **9** | **2031** | **225.7 ***** | **58.9** |
| **Temperature** | **2** | **5688** | **2843.9 ***** | **742.3** |
| **Genotype*temperature** | **18** | **1627** | **90.4***** | **23.6** |
| **Residuals** | **60** | **230** | **3.8** |  |
| **Relative water content** | | | | |
| **Genotype** | **9** | **2057** | **228.6***** | **77.7** |
| **Temperature** | **2** | **4303** | **2151.3***** | **731.8** |
| **Genotype*temperature** | **18** | **719** | **39.9***** | **13.5** |
| **Residuals** | **60** | **176** | **2.9** |  |
| **Chlorophyll florescence** | | | |  |
| **Genotype** | **9** | **0.005488** | **00.000610 ***** | **4.5** |
| **Temperature** | **2** | **0.00684** | **0.003424 ***** | **25.6** |
| **Genotype*temperature** | **18** | **0.00416** | **0.000231 ***** | **1.7** |
| **Residuals** | **30** | **0.00400** | **0.000134** |  |
| **Canopy temperature** | | | | |
| **Genotype** | **9** | **100.5** | **11.2***** | **21.4** |
| **Temperature** | **2** | **1333.3** | **666.6***** | **1279.3** |
| **Genotype*temperature** | **18** | **121.5** | **6.8***** | **12.9** |
| **Residuals** | **60** | **31.3** | **0.5** |  |

Note: ****; significant at **p* = 0.05, ***p* = 0.01 and ****p* = 0.001; ns, not significant.

**Table S3** ANOVA for important photosynthetic and gaseous parameters in contrasting set of cucumber genotypes

| **Source of variation** | **Df** | **Sum Sq** | **Mean Sq** | **F value** |
| --- | --- | --- | --- | --- |
| **Net photosynthesis** | | | | |
| **Genotype** | **9** | **210.3** | **23.4***** | **186.9** |
| **Temperature** | **2** | **781.7** | **390.8***** | **3126.9** |
| **Genotype*temperature** | **18** | **282.9** | **15.7 ***** | **125.7** |
| **Residuals** | **30** | **3.7** | **0.1** |  |
| **Stomatal conductance** | | | | |
| **Genotype** | **9** | **0.8056** | **0.0895 ***** | **62.1** |
| **Temperature** | **2** | **1.8161** | **0.9080***** | **630.3** |
| **Genotype*temperature** | **18** | **1.2406** | **0.0689***** | **47.8** |
| **Residuals** | **30** | **0.0432** | **0.0014** |  |
| **Internal C**$\mathbf{O}_{\mathbf{2}}$ **concentration** | | | | |
| **Genotype** | **9** | **103570** | **11508***** | **412.2** |
| **Temperature** | **2** | **1590143** | **795071***** | **28482.4** |
| **Genotype*temperature** | **18** | **46557** | **2586***** | **92.6** |
| **Residuals** | **30** | **837** | **28** |  |
| **Transpiration rate** | | | |  |
| **Genotype** | **9** | **19.5** | **2.18 ***** | **8.6** |
| **Temperature** | **2** | **82.3** | **41.15***** | **162.6** |
| **Genotype*temperature** | **18** | **51.7** | **2.88 ***** | **11.3** |
| **Residuals** | **30** | **7.5** | **0.25** |  |

Note: ****; significant at **p* = 0.05, ***p* = 0.01 and ****p* = 0.001; ns, not significant.

**Table S4** ANOVA for important morphological traits in contrasting set of cucumber genotypes

| **Source of variation** | **Df** | **Sum Sq** | **Mean Sq** | **F value** |
| --- | --- | --- | --- | --- |
| **Shoot length** | | | | |
| **Genotype** | **9** | **2429** | **270***** | **10.2** |
| **Temperature** | **1** | **3573** | **3573***** | **136.1** |
| **Genotype*temperature** | **9** | **2576** | **286***** | **10.9** |
| **Residuals** | **40** | **1050** | **26** |  |
| **Fresh weight** | | | | |
| **Genotype** | **9** | **2699** | **300***** | **652.7** |
| **Temperature** | **1** | **15005** | **15005 ***** | **32662.2** |
| **Genotype*temperature** | **9** | **2439** | **271***** | **589.9** |
| **Residuals** | **40** | **18** | **0** |  |
| **Dry weight** | | | | |
| **Genotype** | **9** | **30.9** | **3.441***** | **150.6** |
| **Temperature** | **1** | **17.4** | **17.477***** | **764.9** |
| **Genotype*temperature** | **9** | **3.1** | **0.355***** | **15.5** |
| **Residuals** | **40** | **0.9** | **0.023** |  |

Note: ****; significant at **p* = 0.05, ***p* = 0.01 and ****p* = 0.001; ns, not significant.

**Table S5** ANOVA for important biochemical traits in contrasting set of cucumber genotypes

| **Source of variation** | **Df** | **Sum Sq** | **Mean Sq** | **F value** |
| --- | --- | --- | --- | --- |
| **Proline** | | | | |
| **Genotype** | **9** | **452.1** | **50.24***** | **528.0** |
| **Temperature** | **2** | **197.0** | **98.51***** | **1035.5** |
| **Genotype*temperature** | **18** | **310.7** | **17.26***** | **181.4** |
| **Residuals** | **60** | **5.7** | **0.10** |  |
| **Super oxide dismutase** | | | | |
| **Genotype** | **9** | **6694** | **743.7***** | **619.4** |
| **Temperature** | **2** | **5571** | **2785.3***** | **2319.5** |
| **Genotype*temperature** | **18** | **9307** | **517.0***** | **430.6** |
| **Residuals** | **60** | **72** | **1.2** |  |
| **Catalase** | | | | |
| **Genotype** | **9** | **436.8** | **48.53 ***** | **861.5** |
| **Temperature** | **2** | **279.1** | **139.57 ***** | **2477.6** |
| **Genotype*temperature** | **18** | **179.9** | **9.99 ***** | **177.4** |
| **Residuals** | **60** | **3.4** | **0.06** |  |
| **Guaiacol Peroxidase content** | | | |  |
| **Genotype** | **9** | **1838.4** | **204.3 ***** | **1571.9** |
| **Temperature** | **2** | **1321.3** | **660.7 ***** | **5084.0** |
| **Genotype*temperature** | **18** | **1011.1** | **56.2***** | **432.3** |
| **Residuals** | **60** | **7.8** | **0.1** |  |
| **Malondialdehyde content** | | | | |
| **Genotype** | **9** | **1429.3** | **158.8 ***** | **747.7** |
| **Temperature** | **2** | **2532.8** | **1266.4 ***** | **5962.3** |
| **Genotype*temperature** | **18** | **669.1** | **37.2 ***** | **175.0** |
| **Residuals** | **60** | **12.7** | **0.2** |  |
| **Protein** | | | | |
| **Genotype** | **9** | **47.4** | **5.276 ***** | **2018.3** |
| **Temperature** | **2** | **4.7** | **2.393 ***** | **915.3** |
| **Genotype*temperature** | **18** | **30.3** | **1.688***** | **645.9** |
| **Residuals** | **60** | **0.1** | **0.003** |  |
| **Ascorbate peroxidase** | | | | |
| **Genotype** | **9** | **69.2** | **7.692***** | **630.6** |
| **Temperature** | **2** | **1.7** | **0.884***** | **72.5** |
| **Genotype*temperature** | **18** | **10.0** | **0.560***** | **45.9** |
| **Residuals** | **60** | **0.7** | **0.012** |  |
| **Hydrogen peroxidase** | | | | |
| **Genotype** | **9** | **1776.3** | **197.3***** | **652.5** |
| **Temperature** | **2** | **21.6** | **10.8***** | **35.7** |
| **Genotype*temperature** | **18** | **1829.5** | **101.6***** | **336.0** |
| **Residuals** | **60** | **18.1** | **0.302** |  |

Note: ****; significant at **p* = 0.05, ***p* = 0.01 and ****p* = 0.001; ns, not significant.
