# Supplementary material for: Elucidating the role of key physio-biochemical traits and molecular network conferring heat stress tolerance in cucumber
Source: Front Plant Sci. 2023 Feb 20;14:1128928. doi: 10.3389/fpls.2023.1128928 (PMC9990136; doi:10.3389/fpls.2023.1128928)
Supplement: Supplementary file 2 [file DataSheet_2.docx]

**
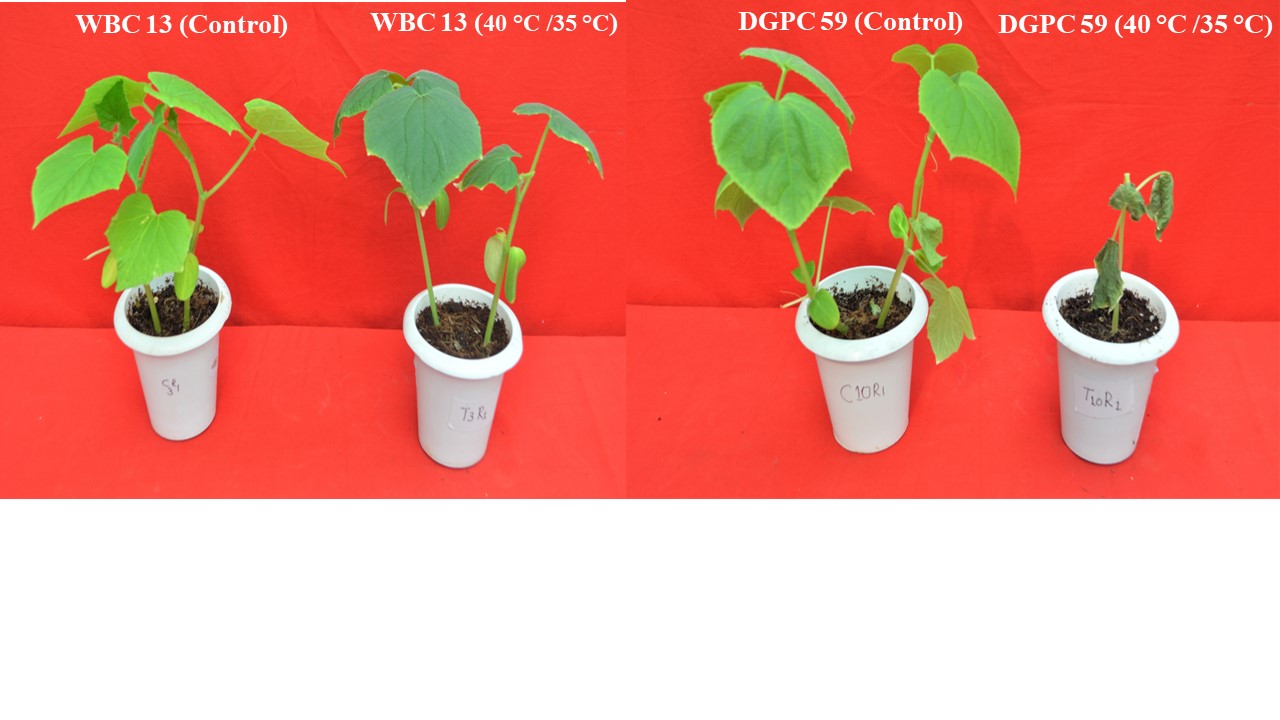
**

**Supplementary Figure 1** Heat stress response of two contrasting cucumber genotype, WBC-13 and DGPC-59 at high stress conditions 40°C/35°C.


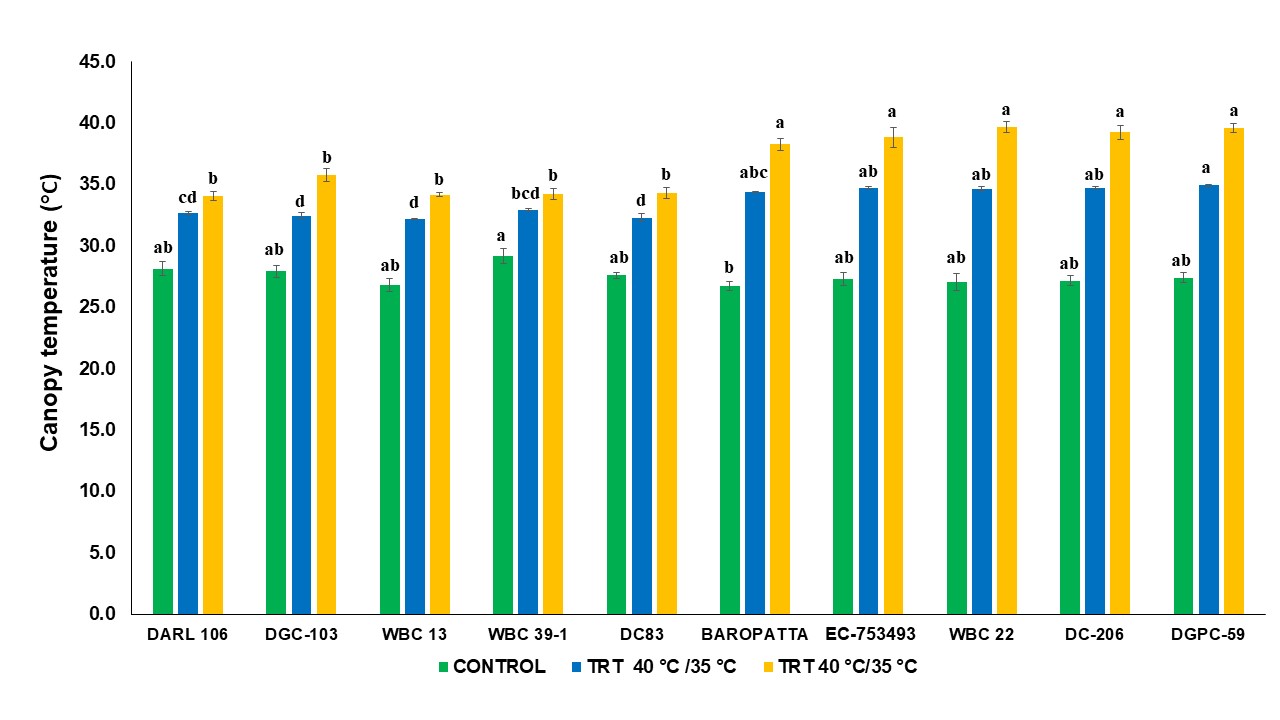
**Supplementary Figure 2** Canopy temperature (means± SE) in cucumber under in control, moderate temperature treatment (35 °C /30 °C) and high temperature treatment (40 °C /35 °C). Different letters indicate significant differences in values at *p<* 0.05 level (Tukey’s HSD Test). Comparisons have made among the 10 genotypes under different conditions. Similar letter within the same condition indicated no significant difference.


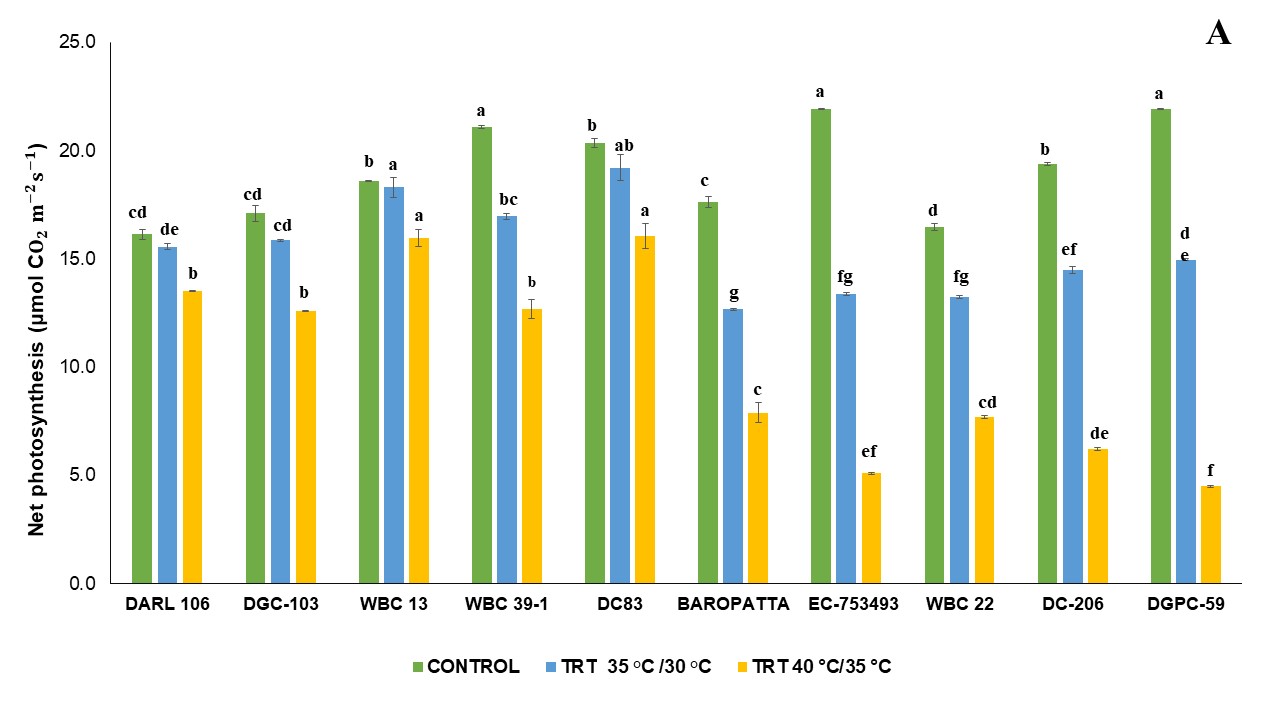


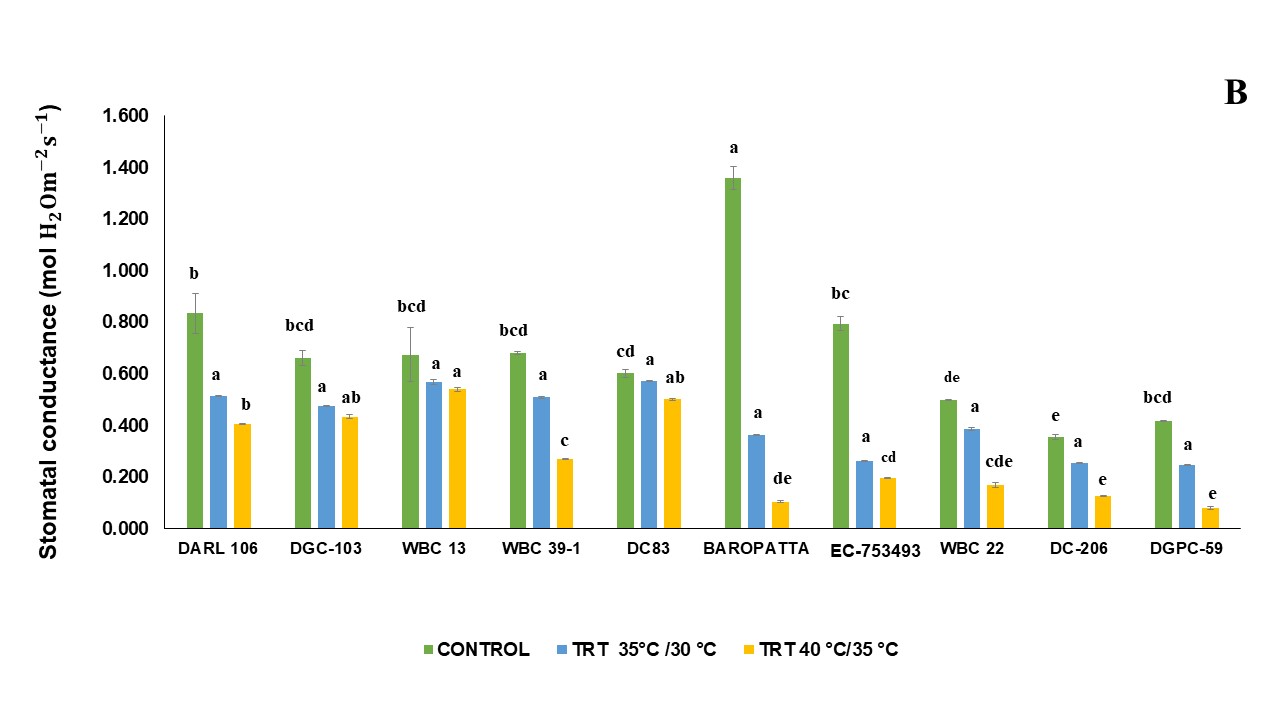


**Supplementary Figure 3** A. Net photosynthesis B. stomatal conductance (means± SE) in cucumber under in control, moderate temperature treatment (35 °C /30 °C) and high temperature treatment (40 °C /35 °C). Different letters indicate significant differences in values at *p<* 0.05 level (Tukey’s HSD Test). Comparisons have made among the 10 genotypes under different conditions. Similar letter within the same condition indicated no significant difference.


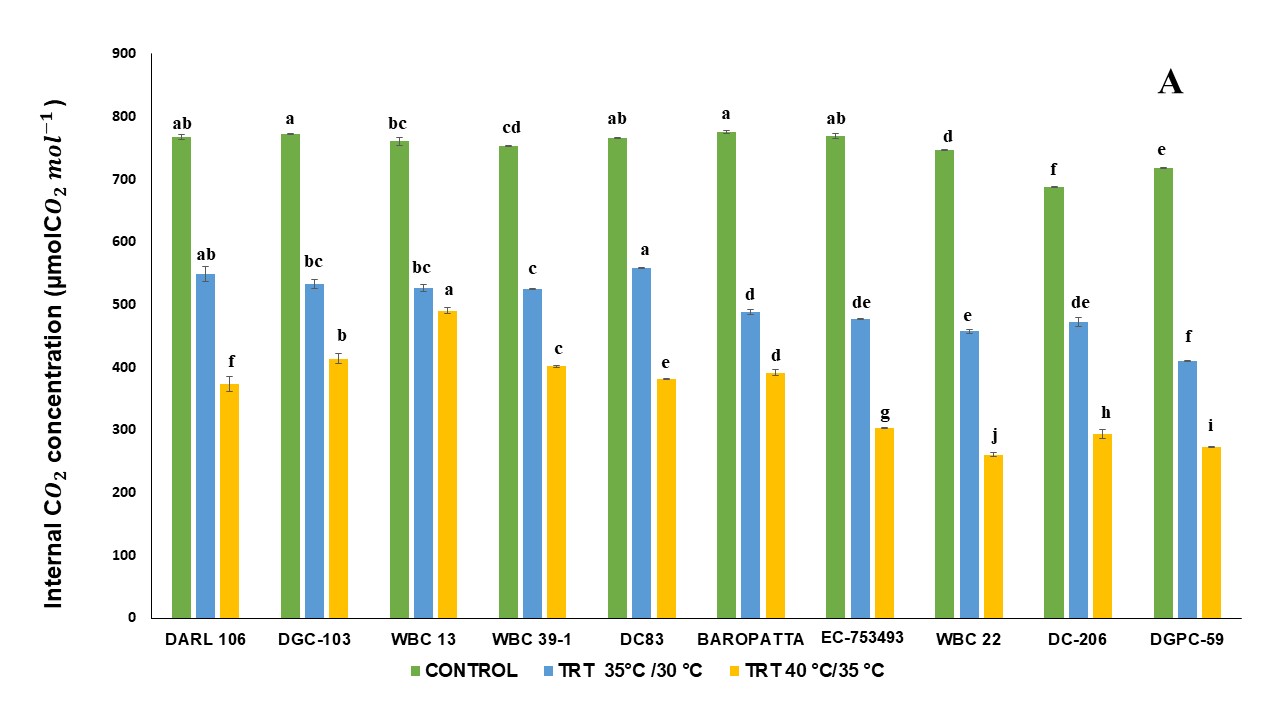


**
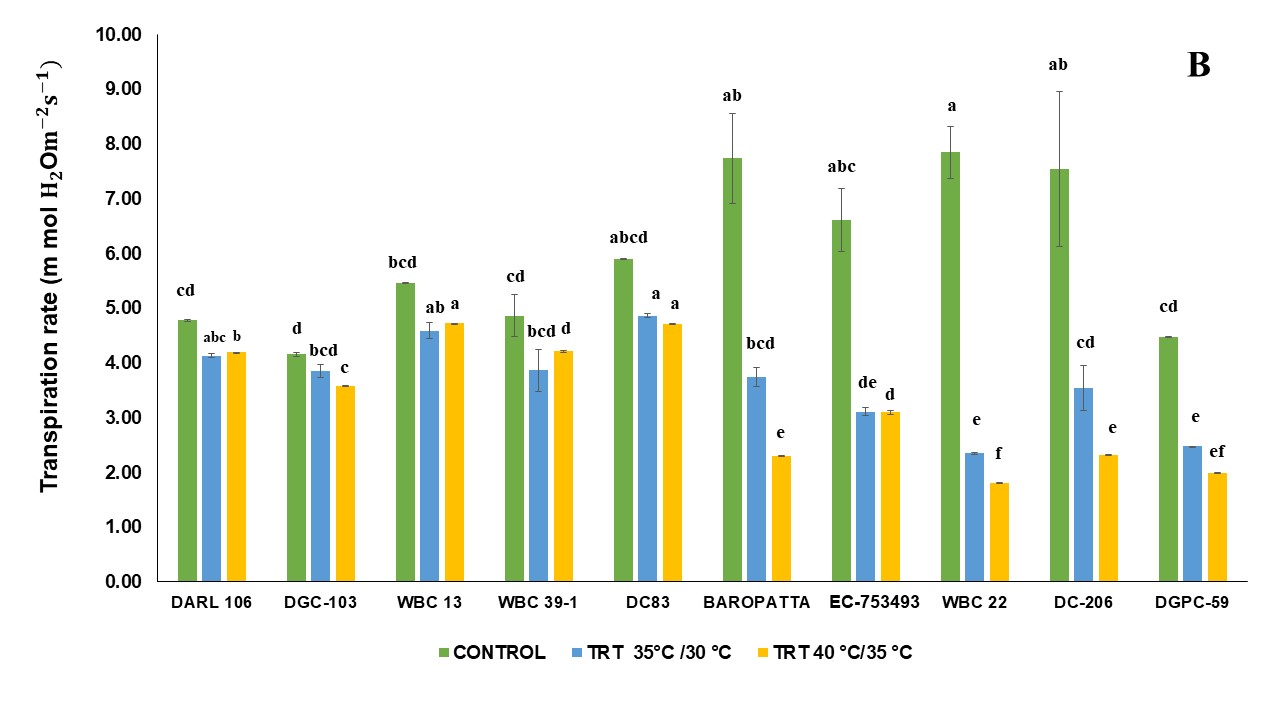
**

**Supplementary Figure 4** A. Internal C$O_{2}$ concentration B. Transpiration rate (means± SE) in cucumber under in control, moderate temperature treatment (35 °C /30 °C) and high temperature treatment (40 °C /35 °C). Different letters indicate significant differences in values at *p<* 0.05 level (Tukey’s HSD Test). Comparisons have made among the 10 genotypes under different conditions. Similar letter within the same condition indicated no significant difference.


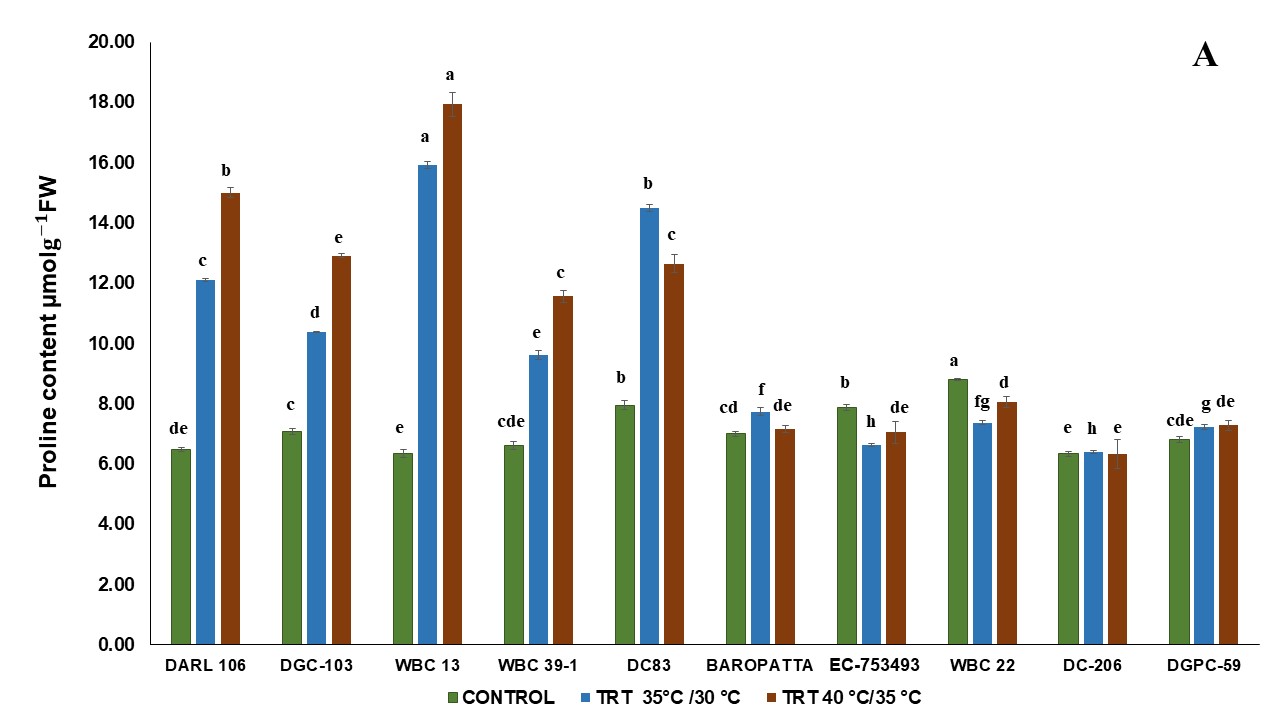


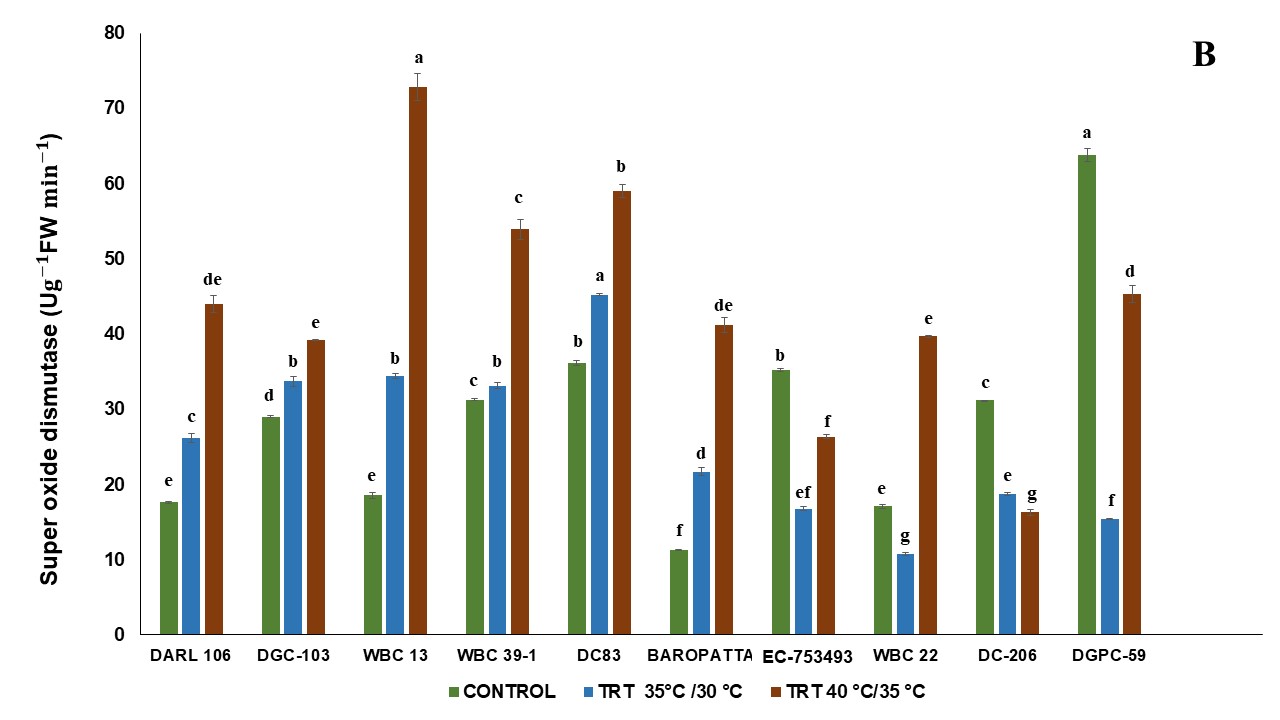


**Supplementary Figure 5** A. Proline B. Super oxide dismutase (means± SE) in cucumber under in control, moderate temperature treatment (35 °C /30 °C) and high temperature treatment (40 °C /35 °C). Different letters indicate significant differences in values at *p<* 0.05 level (Tukey’s HSD Test)


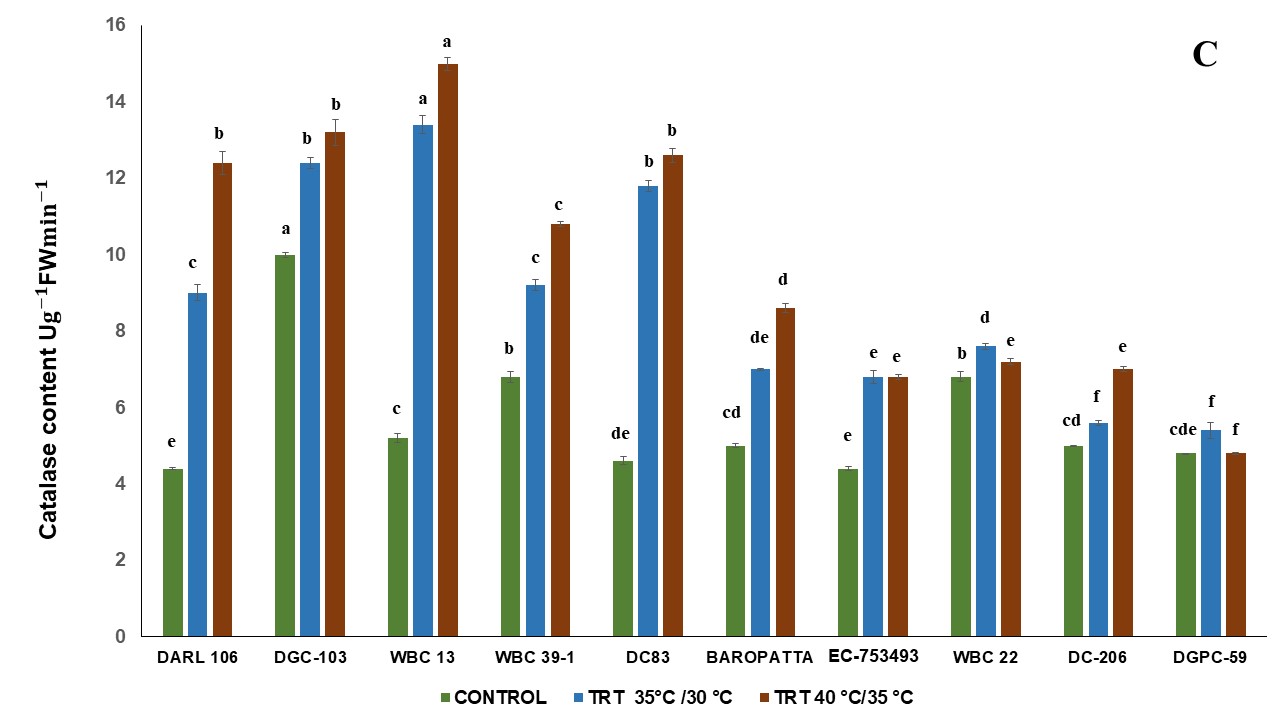


**
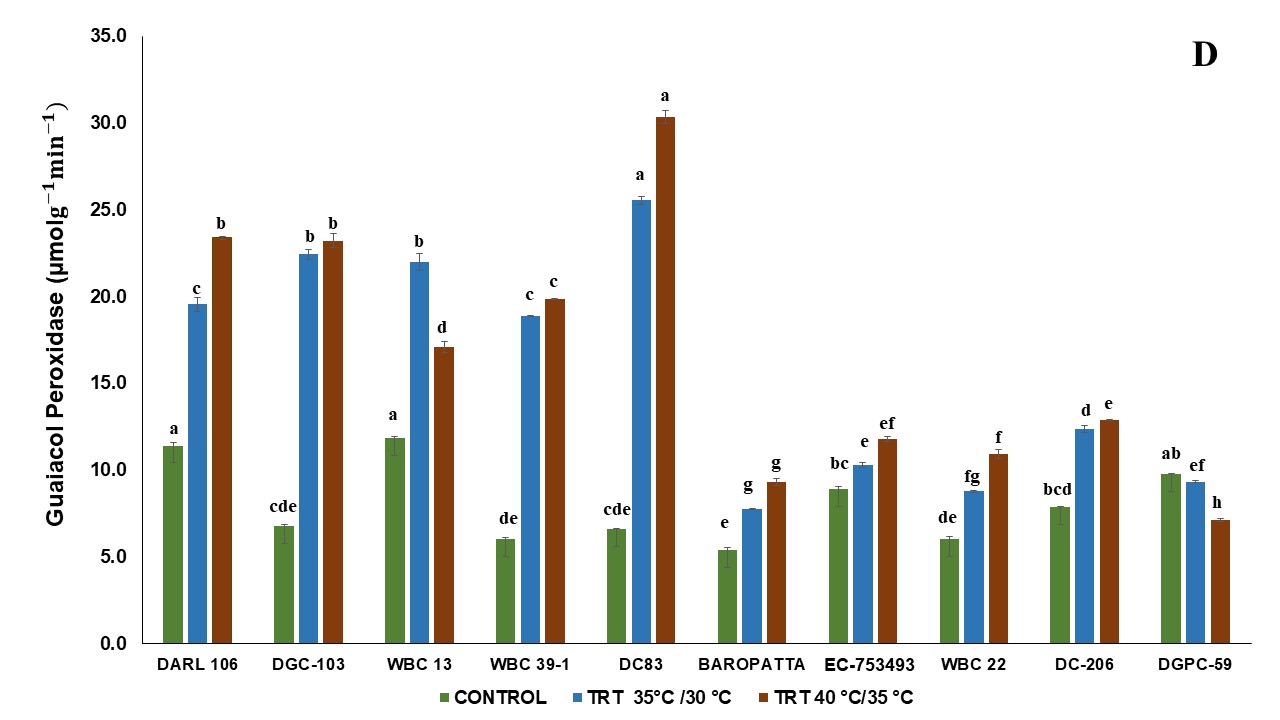
**

**Supplementary Figure 5** C. Catalase D. Guaiacol Peroxidase (means± SE) in cucumber under in control, moderate temperature treatment (35 °C /30 °C) and high temperature treatment (40 °C /35 °C). Different letters indicate significant differences in values at *p<* 0.05 level (Tukey’s HSD Test)


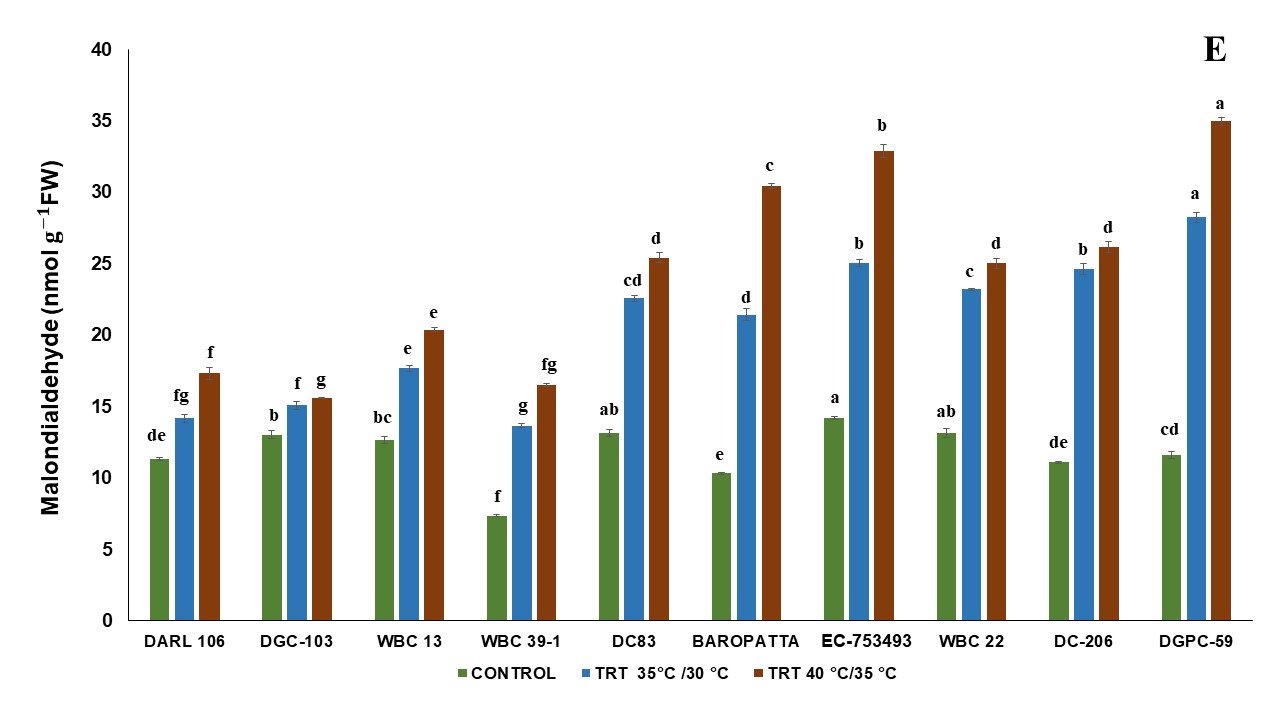


**
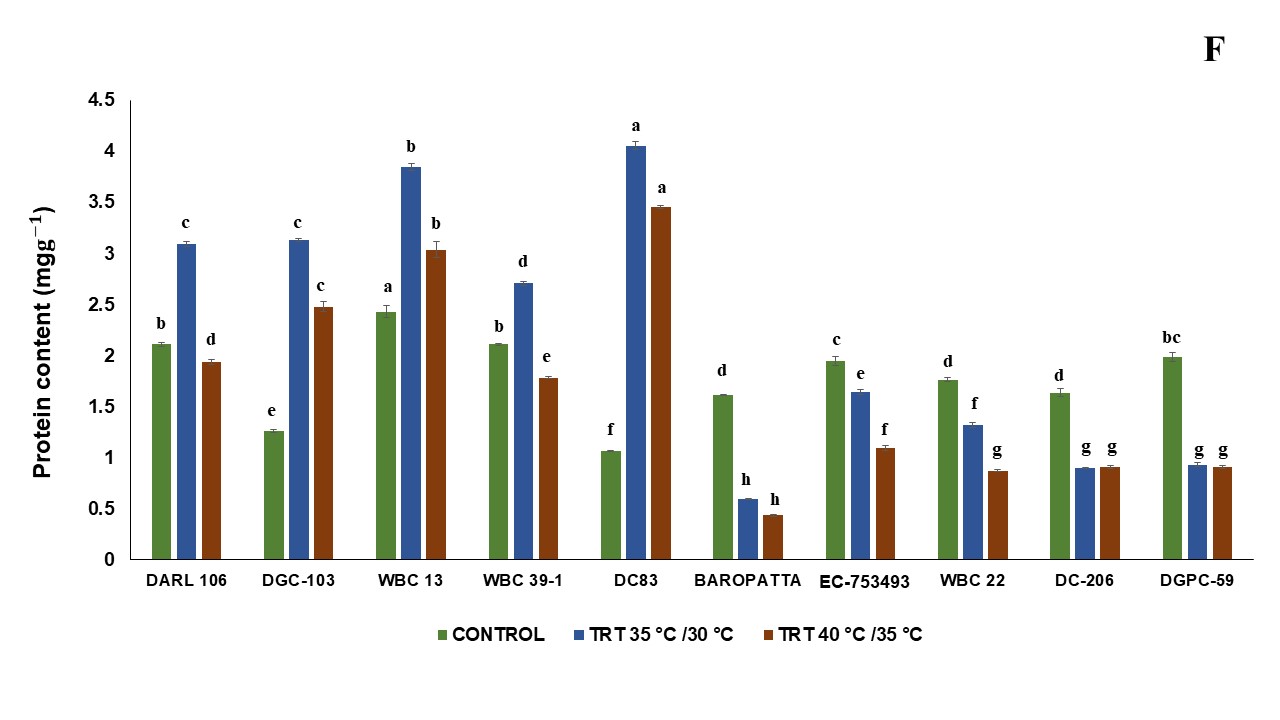
**

**Supplementary Figure 5** E. Malondialdehyde F. Protein (means± SE) in cucumber leaves under in control, moderate temperature treatment (35 °C /30 °C) and high temperature treatment (40 °C /35 °C). Different letters indicate significant differences in values at *p<* 0.05 level (Tukey’s HSD Test)


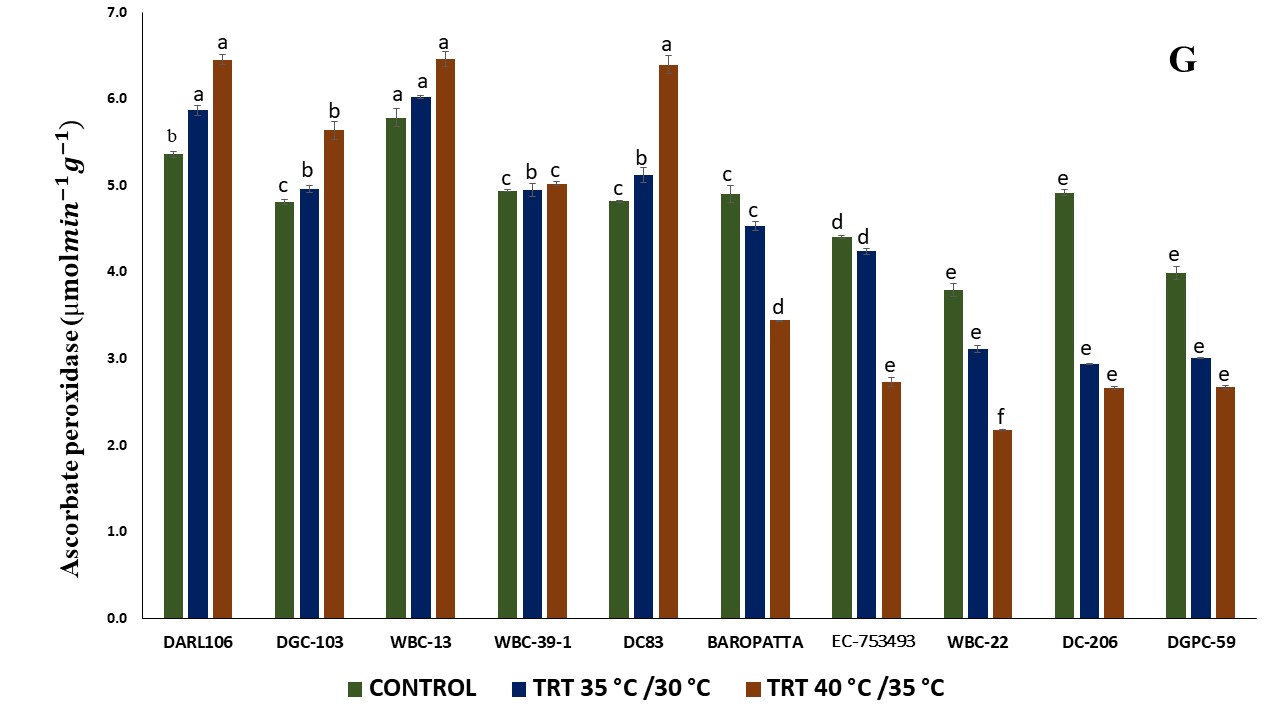


**
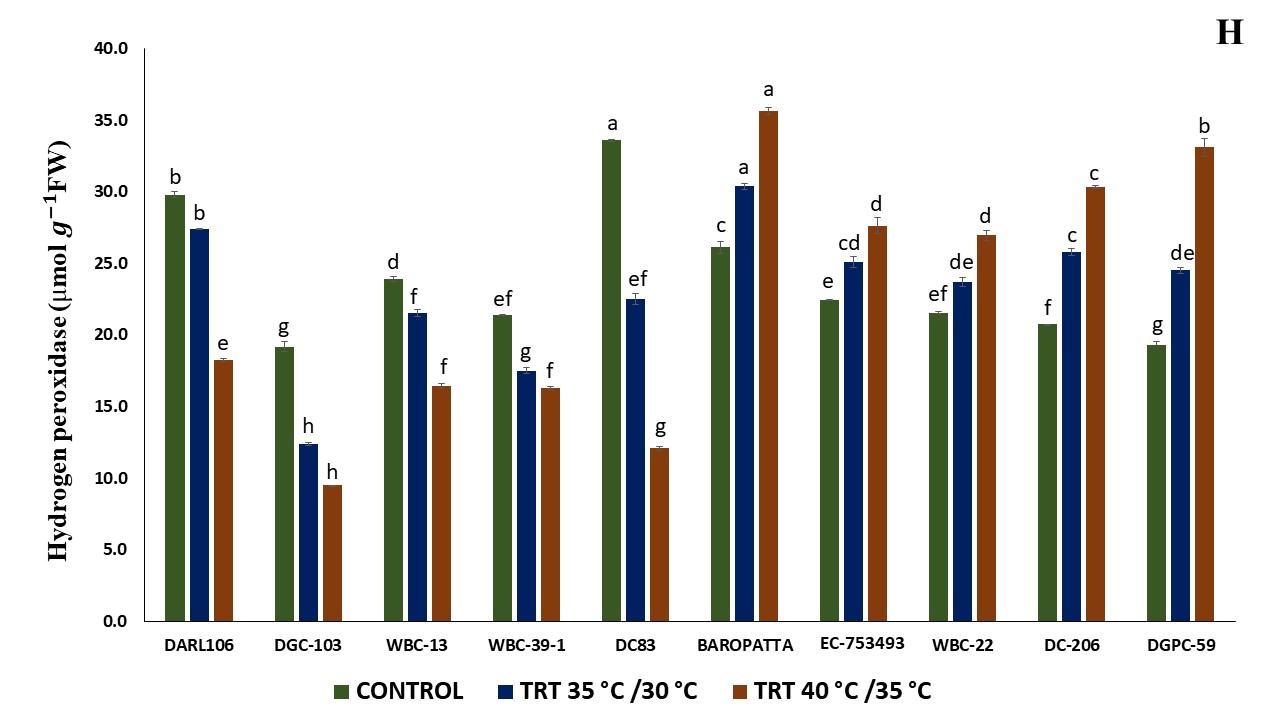
**

**Supplementary Figure 5** G. Ascorbate peroxidase H. Hydrogen peroxidase (means± SE) in cucumber leaves under in control, moderate temperature treatment (35 °C /30 °C) and high temperature treatment (40 °C /35 °C). Different letters indicate significant differences in values at *p<* 0.05 level (Tukey’s HSD Test)
